# Translational Feasibility of Lumbar Puncture for Intrathecal AAV Administration

**DOI:** 10.1016/j.omtm.2020.04.012

**Published:** 2020-04-18

**Authors:** Christian Hinderer, Nathan Katz, Cecilia Dyer, Tamara Goode, Julia Johansson, Peter Bell, Laura Richman, Elizabeth Buza, James M. Wilson

**Affiliations:** 1 Gene Therapy Program, Perelman School of Medicine, University of Pennsylvania, Philadelphia, PA, USA

**Keywords:** AAV, central nervous system, intrathecal, cerebrospinal fluid, lumbar puncture, cisterna magna

## Abstract

Preclinical studies have demonstrated that a single injection of an adeno-associated virus (AAV) vector into the cerebrospinal fluid (CSF) can achieve widespread gene transfer throughout the central nervous system. Successfully translating this approach to humans requires identifying factors that influence AAV distribution in the CSF so that optimal parameters can be replicated in the clinic. In the context of developing a motor neuron-targeted gene therapy for spinal muscular atrophy, we conducted studies in nonhuman primates to evaluate the impact of injection volume on spinal cord transduction after AAV delivery via lumbar puncture. Lumbar injection of an AAVhu68 vector targeted motor neurons throughout the spinal cord, but only in juvenile nonhuman primates administered large injection volumes, equivalent to about half of the total CSF volume. Upon repeating this study with clinically relevant injection volumes and larger animals, we found that lumbar puncture failed to achieve significant transduction of the spinal cord. In contrast, vector administered into the cisterna magna distributed reproducibly throughout the spinal cord in both juvenile and adult animals. These findings highlight the challenges of translating AAV delivery via lumbar puncture to humans and suggest that delivery into the cisterna magna may represent a more feasible alternative.

## Graphical Abstract

**Figure:**
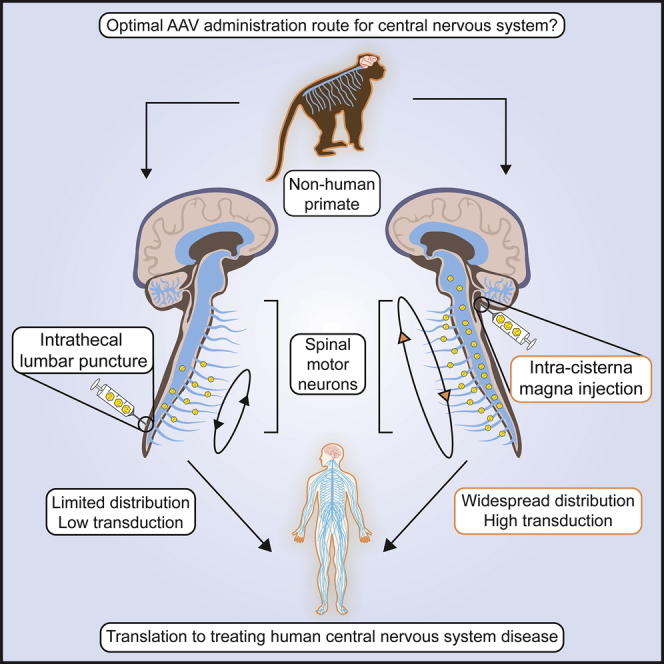

## Teaser

Utilizing a translatable, single-injection approach is crucial for developing successful gene therapies targeting central nervous system diseases. Lumbar puncture administration of clinically relevant vector doses and injection volumes failed to achieve significant spinal cord transduction in nonhuman primates. However, intra-cisterna magna injection achieved reproducible, widespread transduction of spinal motor neurons.

## Introduction

Adeno-associated virus (AAV)-mediated gene transfer has demonstrated the potential for long-term expression of a transgene in the human brain with an acceptable safety profile.^1^, ^2^, ^3^ Most evidence of persistent gene transfer has been limited to clinical trials in which the vector was injected directly into the brain parenchyma.^1^, ^2^, ^3^ Although this approach is promising for some diseases, intraparenchymal injection is an invasive procedure that results in limited distribution of the vector beyond the injection site, thus making it unsuitable for many applications.^3^^,^^4^ Expanding the potential of AAV gene therapy to new targets will require the identification of capsids and delivery methods that can broadly target cells relevant to each disease. Intravenous AAV delivery offers a noninvasive alternative capable of broad vector distribution. When injected intravenously at high doses, AAV serotype 9 (AAV9) can efficiently transduce primary sensory neurons in the dorsal root ganglia and lower motor neurons in the spinal cord and brainstem.^5^ The latter finding led to the development of Zolgensma (onasemnogene abeparvovec-xioi), an intravenous AAV9 gene therapy for the motor neuron disease spinal muscular atrophy (SMA).^6^ Although delivering AAV9 intravenously can efficiently target motor and sensory neurons that project to the periphery, brain transduction is comparatively modest with this approach.^7^^,^^8^ Clinical applications of intravenous delivery are limited by pre-existing AAV neutralizing antibodies, manufacturing challenges that arise from the large required doses, and systemic toxicity associated with these high doses.^5^ AAV delivery into the cerebrospinal fluid (CSF) has several advantages: (1) it requires much lower vector doses, (2) it is unaffected by serum neutralizing antibodies,^9^, ^10^, ^11^ and (3) it can achieve broad transduction in the brain and spinal cord with a single injection.^8^, ^9^, ^10^ Transduction in the brain is diffuse, with only a small percentage of cells expressing the transgene; however, certain cell populations such as the lower motor neurons in the spinal cord are transduced at high frequencies.^8^

In large-animal studies, transduction of the lower motor neurons has been demonstrated after AAV delivery into the CSF via the lateral cerebral ventricles, cisterna magna, or lumbar cistern.^8^^,^^12^ Injection via lumbar puncture has clear advantages given that this is a widely used approach in clinical practice. However, we and others have previously demonstrated that in contrast with intra-cisterna magna (ICM) injection, lumbar puncture results in a dramatically lower vector distribution to the brain and cervical spinal cord of nonhuman primates (NHPs).^8^^,^^13^^,^^14^ Others have also observed limited distribution of AAV from a lumbar puncture in pigs.^15^ In contrast, Meyer et al.^16^ demonstrated efficient transduction of the brain and spinal cord after AAV administration to NHPs through a lumbar puncture. The authors showed that transduction could be further increased by placing animals in the Trendelenburg position, but even without this maneuver, transduction was significantly higher than others have observed following lumbar delivery. Meyer et al.^16^ utilized animals that were substantially smaller and younger than those we employed in our studies, and also mixed the vector with contrast material, resulting in a different formulation and a larger injection volume. We, therefore, explored how these variables impact distribution of an AAV vector from a lumbar puncture to determine whether changes to the procedure could allow a lumbar intrathecal approach to be translated to humans.

## Results

**Figure 1:**
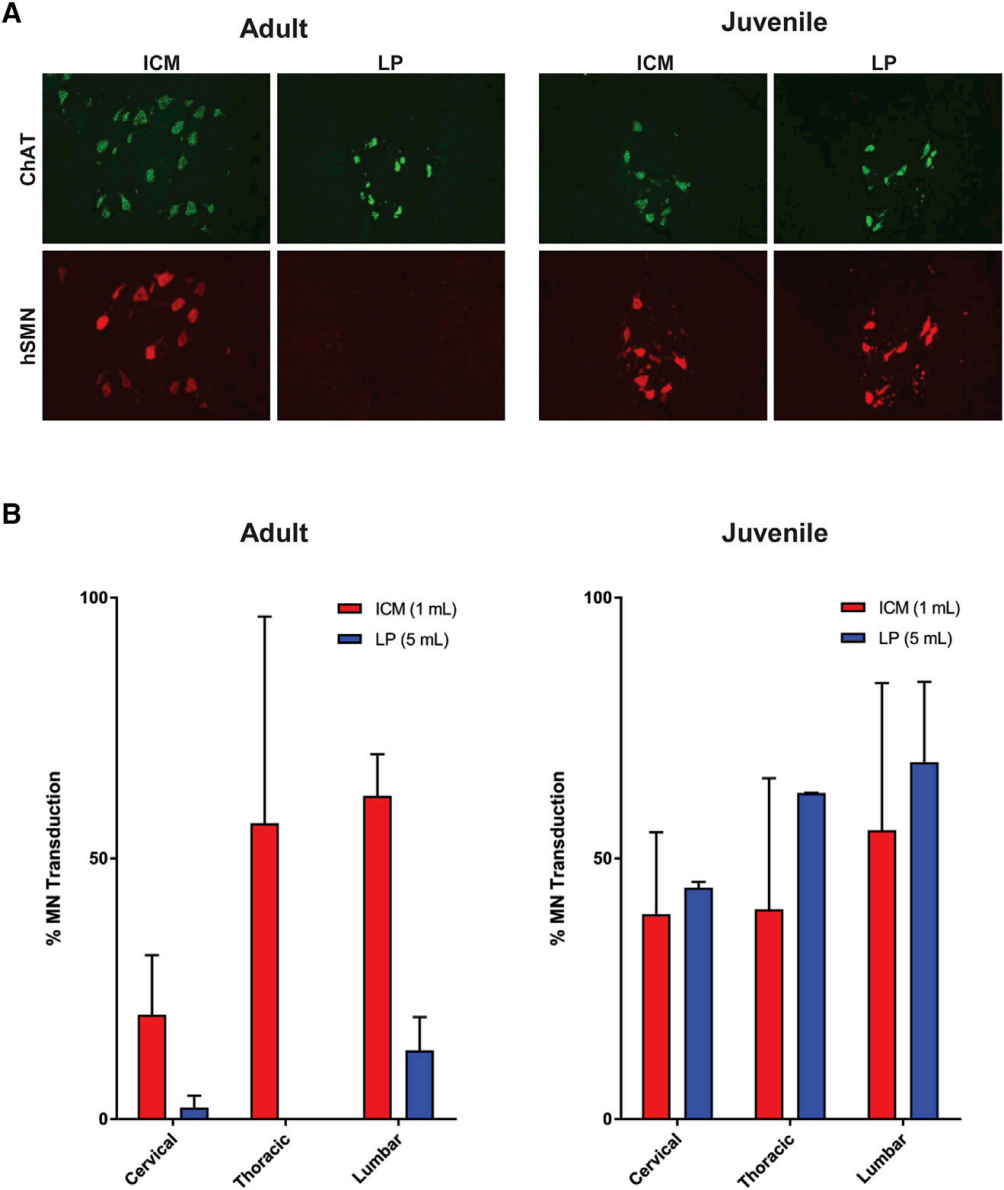
Motor Neuron Transduction following AAVhu68 Administration via Intra-cisterna Magna (ICM) Injection or Lumbar Puncture (LP) to Adult and Juvenile NHPs Adult (3- to 6-year-old) and juvenile (12- to 14-month-old) rhesus macaques were administered 3 × 10^13^ GC of an AAVhu68 vector expressing human SMN from a chicken beta-actin promoter. The vector was injected into the cisterna magna (ICM) in a volume of 1 mL or via LP in a volume of 5 mL. Animals were necropsied 28 days after vector administration. We performed *in situ* hybridization on spinal cord sections using probes for the human SMN transgene and choline acetyltransferase (A). We quantified the percentage of ChAT^+^ motor neurons expressing human SMN in spinal cord sections from cervical, thoracic, and lumbar levels (B). Error bars = standard error of the mean (SEM).

We first attempted to replicate the prior report^16^ of efficient motor neuron transduction in juvenile animals by using a high-volume lumbar AAV injection. In this previous study, the administered injection was a vector mixed with contrast material, which resulted in a higher volume and an increased density.^16^ We tested whether a large injection volume without contrast could replicate these findings. Juvenile (12- to 14-month-old) rhesus macaques were administered 3 × 10^13^ genome copies (GC) of an AAVhu68 vector expressing a human survival of motor neuron (SMN) transgene either via lumbar puncture (5 mL volume) or ICM injection (1 mL volume; see Table 1). *In situ* hybridization (ISH) for the transgene mRNA revealed extensive transduction of motor neurons throughout the spinal cord in both the lumbar puncture and ICM groups (Figure 1). In contrast, when we performed the same comparison in adult animals, only ICM delivery resulted in significant motor neuron transduction.

**Table 1:** Study Design to Compare AAVhu68 Administration via Intra-cisterna Magna (ICM) Injection or Lumbar Puncture (LP) in Adult and Juvenile Rhesus Macaques

Age	Route of Administration	Dose Volume	Number of Animals
Adult (3–6 years)	ICM	1 mL	3
	LP	5 mL	4
Juvenile (12–14 months)	ICM	1 mL	3
	LP	5 mL	2

Macaques were administered 3 × 10^13^ GC of an AAVhu68 vector expressing a human SMN transgene. The study duration was 28 days.

**Figure 2:**
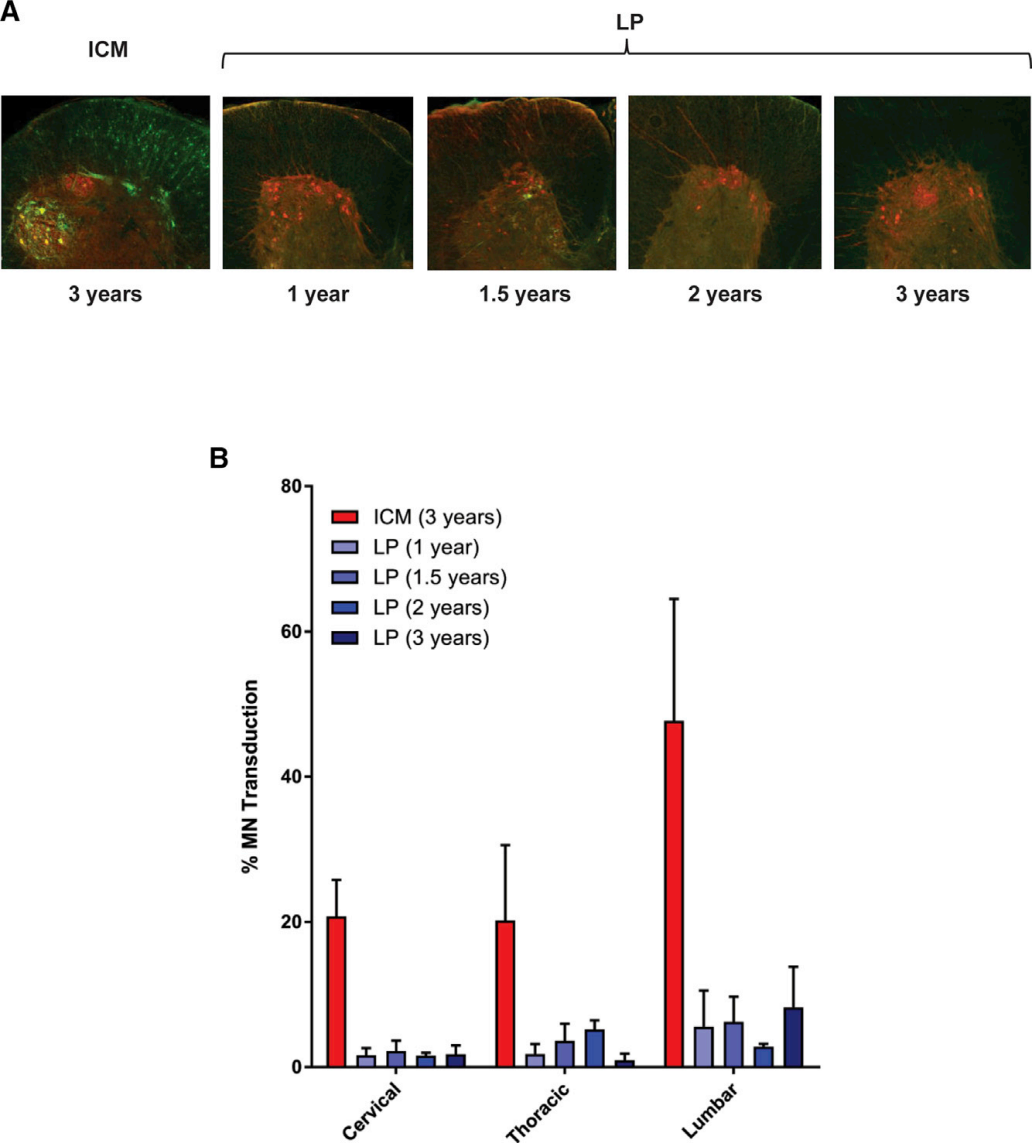
Motor Neuron Transduction following ICM or LP AAVhu68 Administration to NHPs of Different Ages Using Clinically Relevant Injection Volumes Rhesus macaques ranging in age from 1 to 3 years were administered 1.5 × 10^13^ GC of an AAVhu68 vector expressing GFP via LP in a volume of 0.7 mL. A control group of 3-year-old NHPs was administered the same vector and dose volume via ICM injection. Animals were necropsied 21 days after vector administration. We performed immunostaining on spinal cord sections using antibodies against ChAT (red) and GFP (green) (A). We quantified the percentage of ChAT^+^ motor neurons expressing GFP in spinal cord sections from cervical, thoracic, and lumbar levels (B). Error bars = SEM.

Widespread motor neuron transduction after lumbar AAV injection in juvenile, but not adult, NHPs could be explained by mechanical factors, with the large injection volume coupled with the animal’s small size and CSF volume driving cranial diffusion of the bolus. Alternatively, this finding could be explained by intrinsic biological differences between juvenile and adult animals, such as developmental or anatomic differences that alter vector exposure to target cells. We evaluated these possibilities by administering an AAVhu68 vector via lumbar puncture to animals ranging in age from juvenile (12–14 months) to adult (3 years). A control group was composed of 3-year-old macaques that were treated by ICM injection (Table 2). To determine whether the transduction after lumbar administration was solely due to the large volume administered, we selected a clinically relevant injection volume for this study. We estimated a pediatric intrathecal dosing volume of 5 mL in patients with a total CSF volume of about 100 mL. We then scaled the dose volume to the 13 mL CSF volume of an adult rhesus macaque, resulting in a dose volume of 0.7 mL. Vector administration via lumbar puncture in this volume resulted in minimal spinal cord transduction in all animals, regardless of age (Figure 2). By contrast, ICM injection in adult animals using the same dose and volume transduced an average of 20% of motor neurons at the cervical level and 50% at the lumbar level.

**Table 2:** Study Design for Evaluating AAVhu68 Administration via Lumbar Puncture to NHPs of Different Ages Using a Clinically Relevant Injection Volume

Age (years)	Route of Administration	Number of Animals
3	ICM	3
1	LP	4
1.5	LP	4
2	LP	3
3	LP	3

NHPs were administered 1.5 × 10^13^ GC of an AAVhu68 vector expressing a GFP transgene at a dose of 0.7 mL. The study duration was 21 days.

**Figure 3:**
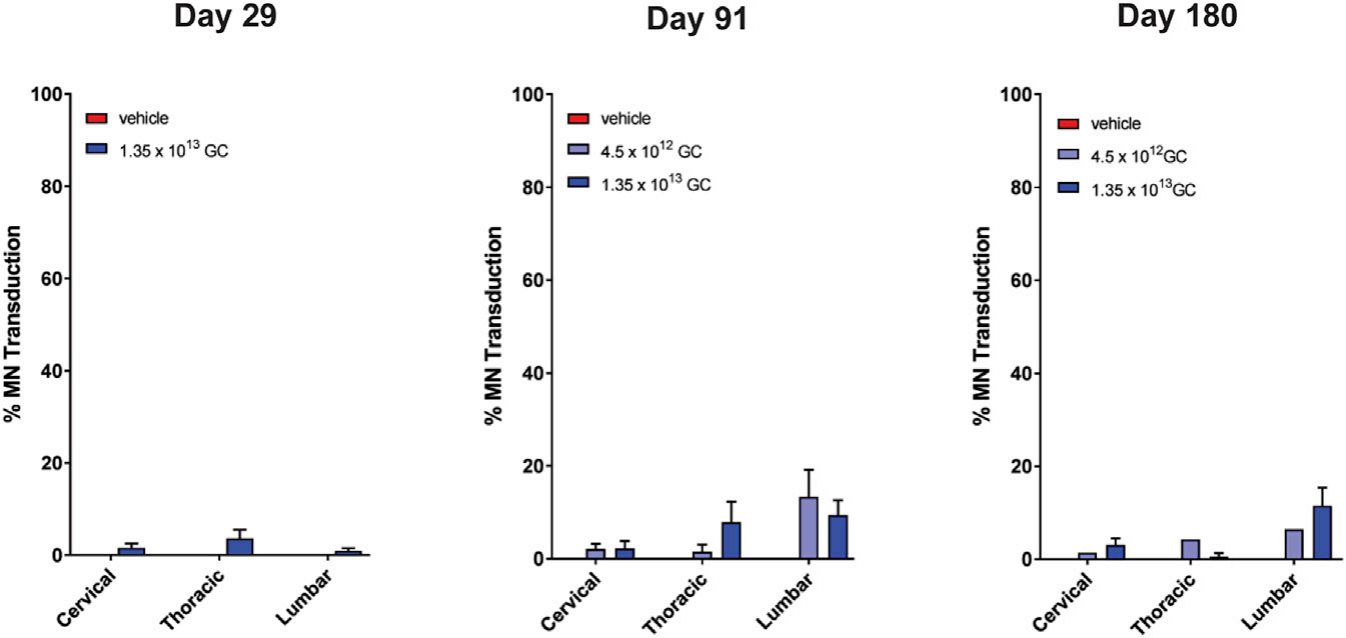
Motor Neuron Transduction in Juvenile NHP Toxicology Study Utilizing LP Injection Juvenile (12- to 14-month-old) rhesus macaques were administered 1.35 × 10^13^ or 4.5 × 10^12^ GC of an AAVhu68 vector expressing human SMN in a volume of 2.5 mL via LP. Control animals were treated with vehicle. Animals were necropsied 29, 91, or 180 days after vector administration. We quantified the percentage of ChAT^+^ motor neurons expressing human SMN in spinal cord sections from cervical, thoracic, and lumbar levels using ISH. Error bars = SEM.

We conducted good laboratory pracitices (GLP)-compliant toxicology studies in both adult and juvenile rhesus macaques using an AAVhu68 vector expressing the human SMN transgene. The study in juvenile animals employed lumbar intrathecal delivery with a dose volume of 2.5 mL and vector doses of 4.5 × 10^12^ and 1.35 × 10^13^ GC. We euthanized animals 1, 3, or 6 months after vector administration and quantified motor neuron transduction using ISH (Figure 3). Transduction was apparent at all time points, with more than 10% of motor neurons transduced in the lumbar segment of some animals. Overall transduction was at least 10-fold lower than observed in the prior juvenile study at a similar time point, potentially due to the lower vector dose and injection volume. The low transduction achieved in this study precluded meaningful analysis of safety.

**Figure 4:**
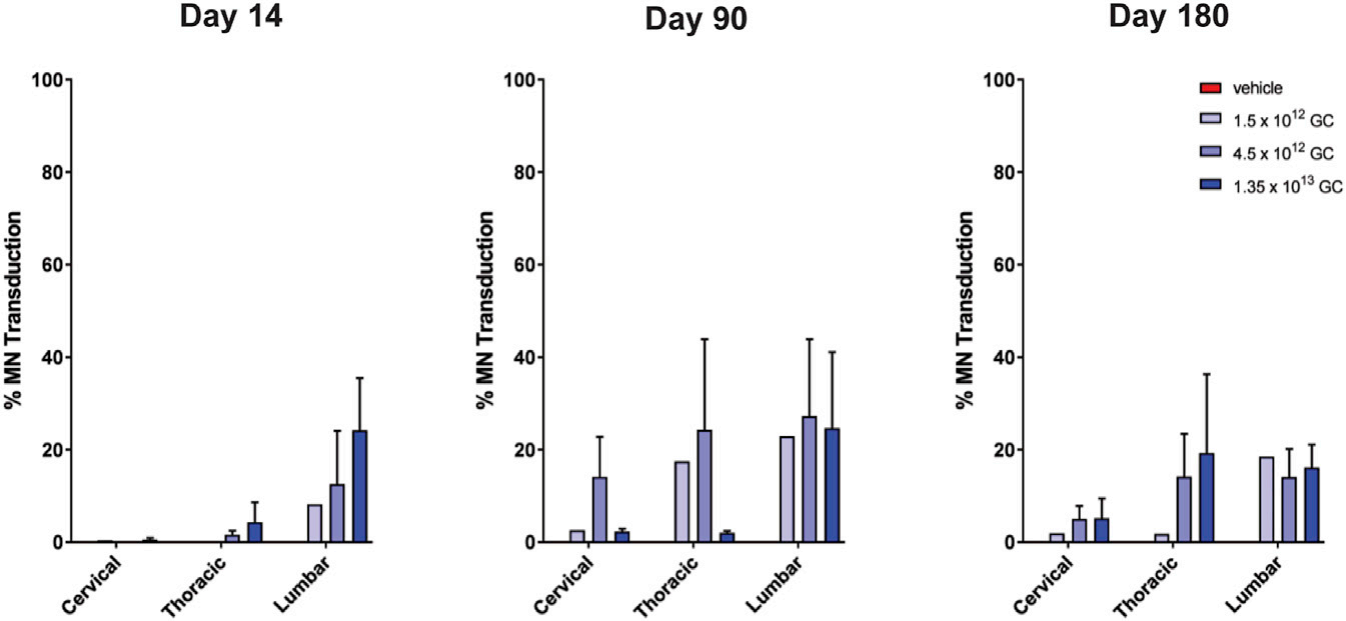
Motor Neuron Transduction in an Adult NHP Toxicology Study Utilizing ICM Injection Adult rhesus macaques were administered 1.35 × 10^13^, 4.5 × 10^12^, or 1.5 × 10^12^ GC (n = 9 per dose) of an AAVhu68 vector expressing human SMN in a volume of 1 mL via ICM injection. Control animals (n = 3) were treated with vehicle. Animals were necropsied 14, 90, or 180 days after vector administration. We quantified the percentage of ChAT^+^ motor neurons expressing human SMN in spinal cord sections from cervical, thoracic, and lumbar levels using ISH. Error bars = SEM.

The toxicology study performed in adult rhesus macaques employed ICM administration and a 1 mL injection volume (Table S1). Transduction of motor neurons was apparent at all necropsy time points, ranging from 14 to 180 days after vector administration (Figure 4). Motor neuron transduction was lower than observed in the prior adult NHP study in which the same vector was administered via an ICM injection at a dose that was roughly 2-fold higher. About 20% of lumbar and thoracic motor neurons were transduced with less transduction in the cervical spinal cord. A dose-response was not clear, and trends were difficult to evaluate due to the modest overall transduction levels. Transgene expression persisted throughout the 6-month study.

Vector DNA was detectable in CSF and peripheral blood; there was a correlation between peak concentrations in CSF and dose (Figure S1). The concentration of vector DNA in CSF rapidly declined following the first evaluated time point (day 7). Vector genomes in blood declined more slowly, which may be attributable to transduction of peripheral blood cells. At 180 days after administration, vector genomes were undetectable in the blood of all animals and were detectable at low levels (51 copies/12 μL) in the CSF of only one animal. Vector genomes were detectable in the urine and feces 5 days after administration; there was a correlation between peak concentrations and dose (Figure S2). Excretion rapidly declined, with most animals exhibiting undetectable levels in both urine and feces by day 28. On day 90, all animals except one had reached undetectable levels of vector DNA in urine. Only one animal in the high-dose cohort had detectable vector DNA in feces (60 copies/μg DNA) on day 90, which was near the limit of detection (50 copies/μg DNA).

Using quantitative PCR, we detected vector genomes at high levels in the brain, spinal cord, dorsal root ganglia, liver, and spleen (see Figure S3), consistent with previous studies of ICM AAV administration.^10^ Lower levels of vector DNA were detectable in most tissues sampled, including skeletal muscle, lung, kidney, lymph nodes, and gonads. The quantity of vector genomes detected in CNS tissues was generally dose dependent. Animals euthanized 90 and 180 days after vector administration exhibited modestly lower vector genome levels in tissues compared with animals euthanized on day 14. This indicates that some vector genomes detected soon after administration do not represent stable transduction. Vector genomes in CNS tissues appeared stable between 90 and 180 days after injection.

We did not detect any dose-limiting toxicity in this study. One animal unexpectedly extended its neck during the ICM injection procedure, resulting in suspected needle penetration of the brainstem. Upon recovery from anesthesia, this animal exhibited mild unilateral weakness. The animal was treated with a short course of prednisone, and the motor deficit resolved within a week of the injection. There were no other clinical abnormalities noted throughout the study. None of the animals exhibited any clinically meaningful changes in hematology, coagulation, or blood and CSF clinical chemistry parameters. One animal treated with the highest vector dose exhibited an asymptomatic self-limited lymphocytic pleocytosis between 28 and 60 days after vector administration (Figure S4). Similar to previous studies of intrathecal AAV administration, all groups exhibited asymptomatic minimal degeneration of dorsal root ganglia sensory neurons and minimal-to-moderate degeneration of their associated axons in the dorsal white matter tracts of the spinal cord with no clear dose dependence (Figures S5 and S6).^10^ These findings emerged on day 14 and developed fully by day 90, with no further progression from days 90 to 180.

Neutralizing antibodies to the AAVhu68 capsid were elicited in all vector-treated animals (Figure S7). Interferon-gamma T cell responses to the capsid and transgene product were detectable in peripheral blood, as well as in lymphocytes harvested from the liver, spleen, and bone marrow of some animals (Figure S8). T cell responses to the AAVhu68 capsid occurred in 12 of 27 vector-treated animals, and 15 of 27 exhibited responses to the human SMN protein, with similar frequencies in all dose cohorts. T cell responses were not associated with abnormal clinical or histological findings. T cell responses to the AAVhu68 capsid were detectable prior to treatment in three animals, potentially due to prior infections with other AAV serotypes with conserved T cell epitopes.

## Discussion

The present studies elucidate key determinants of AAV distribution in CSF and help explain inconsistencies in previous studies. The results of ICM vector administration in NHPs were consistent with our prior studies^8^^,^^10^^,^^17^ and other reports from the literature.^18^ Although we previously reported that lumbar puncture is far less effective than ICM AAV administration in NHPs,^8^ here we were able to reproduce the successful gene transfer to the spinal cord reported by another group by more closely replicating aspects of their model system and injection method.

Following ICM AAV administration, we consistently observed a lumbar-to-cervical gradient of motor neuron transduction despite the greater distance from the lumbar region to the injection site. We hypothesize that this is related to the anatomy of the lumbar motor neurons, the axons of which may be more exposed to CSF due to the long ventral roots of the lumbar spinal cord. In the present studies, we observed a nonlinear dose-response with markedly lower motor neuron transduction when the dose of the human SMN-expressing vector was decreased from 3 × 10^13^ GC in pilot studies to 1.35 × 10^13^ GC in the toxicology study. This observation may have been partly related to the low sensitivity of ISH for detecting transgene expression, as the study conducted with a GFP reporter gene demonstrated higher overall transduction levels. However, our results suggest that relatively high doses, equivalent to an NHP dose of at least 1.5 × 10^13^ GC, may be required to achieve reliable motor neuron transduction, particularly of the cervical spinal cord. ICM vector administration had a similar safety profile to previous studies;^10^^,^^17^ mild transient pleocytosis and minimal asymptomatic degeneration of sensory neurons in the dorsal root ganglia were the only vector-related adverse effects.

Our findings suggest that AAV delivery via lumbar puncture, although feasible in nonclinical studies, will be challenging to translate to humans. Delivery to the entire spinal cord was possible only when the vector was administered in a large volume in small NHPs, indicating that the distribution of the vector was driven by the spread of the injected bolus rather than by circulation of the vector in CSF. Directly replicating this approach in humans may require administering roughly half of the total CSF volume, which is inconsistent with clinical practice and may cause toxicity from rapid changes in intracranial pressure and CSF electrolytes. Moreover, others have demonstrated that cranial spread of a lumbar bolus is impaired if the dura has been punctured in a previous injection attempt.^13^ If administering a one-time therapy relies on a lumbar puncture that is always successful on the first attempt, then an unacceptable level of variability may be apparent in clinical outcomes.

These studies were carried out in the context of developing a gene therapy for SMA. Our analysis was therefore focused on the transduction of motor neurons in the spinal cord, although we expect these findings to be broadly applicable to programs that target the spinal cord or brain. The challenges of AAV distribution to the spinal cord following lumbar injection are likely to be further amplified when the brain is the primary target. Our findings suggest that ICM administration may overcome these delivery challenges.

## Materials and Methods

### Vectors

Vectors were produced by triple transfection of adherent HEK293 cells and purified by ultracentrifugation on an iodixanol gradient (GFP vectors) or by affinity chromatography (SMN vectors) as previously described.^5^

### Animal Procedures

All animal protocols were approved by the Institutional Animal Care and Use Committee of the University of Pennsylvania. Adult NHPs were 3–8 kg at the time of dosing. One-year-old NHPs were 1.5–3 kg at the time of dosing. NHPs were anesthetized with ketamine and dexmedetomidine for all procedures. ICM injection was performed as previously described.^19^ Lumbar puncture was performed under fluoroscopic guidance in anesthetized animals. After inserting a spinal needle into the L4–5 or L5–6 space, we confirmed placement by CSF return and/or by injecting up to 1 mL of contrast material (Iohexol 180). After confirming placement, we injected the vector solution by hand at a rate of approximately 2 mL/min. Complete blood counts and serum chemistry panels were evaluated prior to vector administration, then weekly for 1 month and monthly thereafter. CSF (1 mL) was collected by suboccipital puncture. We performed euthanasia with a pentobarbital overdose.

### Vector Biodistribution and Pharmacokinetics

We quantified the vector genomes in the blood, CSF, urine, feces, and tissue samples by TaqMan PCR as previously described.^10^^,^^17^

### Immunological Assays

Neutralizing antibody assays and ELISPOTs were performed as previously described.^5^

### Histology

We performed tissue fixation and ISH as previously described.^5^ For quantification of motor neuron transduction, two sections from the cervical, thoracic, and lumbar levels were analyzed. The total number of choline acetyltransferase (ChAT)^+^ motor neurons and the number of human SMN^+^/ChAT^+^ cells was determined for each spinal cord level, and mean percent transduction was calculated. For histopathology, we sectioned and stained formalin-fixed, paraffin-embedded tissues with hematoxylin and eosin according to standard protocols. A board-certified veterinary pathologist examined the slides. A second pathologist peer reviewed the slides for the toxicology studies.

## Author Contributions

C.H., N.K., and J.M.W. designed experiments; C.H., N.K., C.D., T.G., J.J., P.B., L.R., and E.B. conducted experiments; C.H. and J.M.W. wrote the paper.

## Conflicts of Interest

J.M.W. is a paid advisor to and holds equity in Scout Bio and Passage Bio; he holds equity in Surmount Bio; he also has a sponsored research agreement with Ultragenyx, Biogen, Janssen, Precision Biosciences, Moderna Inc., Scout Bio, Passage Bio, Amicus Therapeutics, and Surmount Bio, which are licensees of Penn technology. J.M.W. is an inventor on patents that have been licensed to various biopharmaceutical companies and for which he may receive payments. C.H. is an inventor on patents licensed to biopharmaceutical companies and holds equity in Scout Bio.

## Supplemental Information

Document S1. Table S1 and Figures S1–S8

Document S2. Article plus Supplemental Information
