# Supplementary material for: Translational Feasibility of Lumbar Puncture for Intrathecal AAV Administration
Source: Mol Ther Methods Clin Dev. 2020 Apr 18;17:969–74. doi: 10.1016/j.omtm.2020.04.012 (PMC7218226; doi:10.1016/j.omtm.2020.04.012)
Supplement: Document S1. Table S1 and Figures S1–S8 [file mmc1.pdf]

OMTM, Volume *17*

Supplemental Information

Translational Feasibility of Lumbar Puncture for Intrathecal AAV Administration

Christian Hinderer, Nathan Katz, Cecilia Dyer, Tamara Goode, Julia Johansson, Peter Bell, Laura Richman, Elizabeth Buza, and James M. Wilson

Treatment (Dose)	Animal ID	Sex	Necropsy (days post injection)
Vehicle (N/A)	RA2424	Male	14
	RA2456	Male	90±2
	RA2382	Female	180±2
Low dose (1.50 x 10^{12} GC)	RA1156	Male	14
	RA2031	Female	
	RA0704	Female	
	RA0549	Male	90±2
	RA2369	Female	
	RA2412	Female	
	RA2464	Male	180±2
	RA2434	Male	
	RA2413	Female	
Mid-dose (4.50 x 10^{12} GC)	RA2463	Male	14
	RA2433	Male	
	RA2410	Female	
	RA1875	Male	90±2
	RA1853	Male	
	RA2360	Female	
	RA2467	Male	180±2
	RA2426	Male	
	RA2150	Female	
High dose (1.35 x 10^{13} GC)	RA2444	Male	14
	RA2468	Male	
	RA2363	Female	
	RA2457	Male	90±2
	RA2371	Female	
	RA2153	Female	
	RA2452	Male	180±2
	RA2400	Female	
	RA2375	Female	

Supplemental Table 1. Design of toxicology study for ICM administration of AAVhu68 expressing human SMN in adult NHPs.
Animals were randomized to treatment groups. All injections were performed in a total volume of 1 mL.

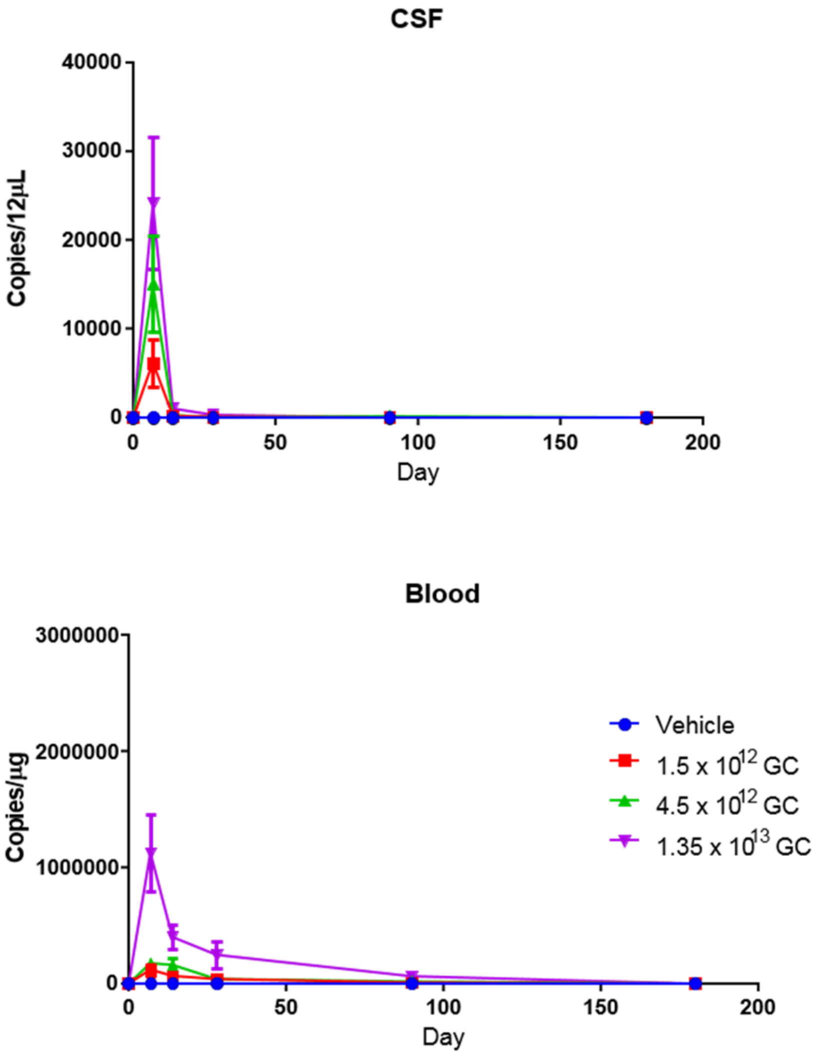

Supplemental Figure 1. Vector Genomes Measured in CSF and Blood Following ICM Administration to NHPs.
Each line represents mean vector genomes detected per microgram DNA (blood) or per 12 µL of CSF for each cohort. Error bars represent SEM.

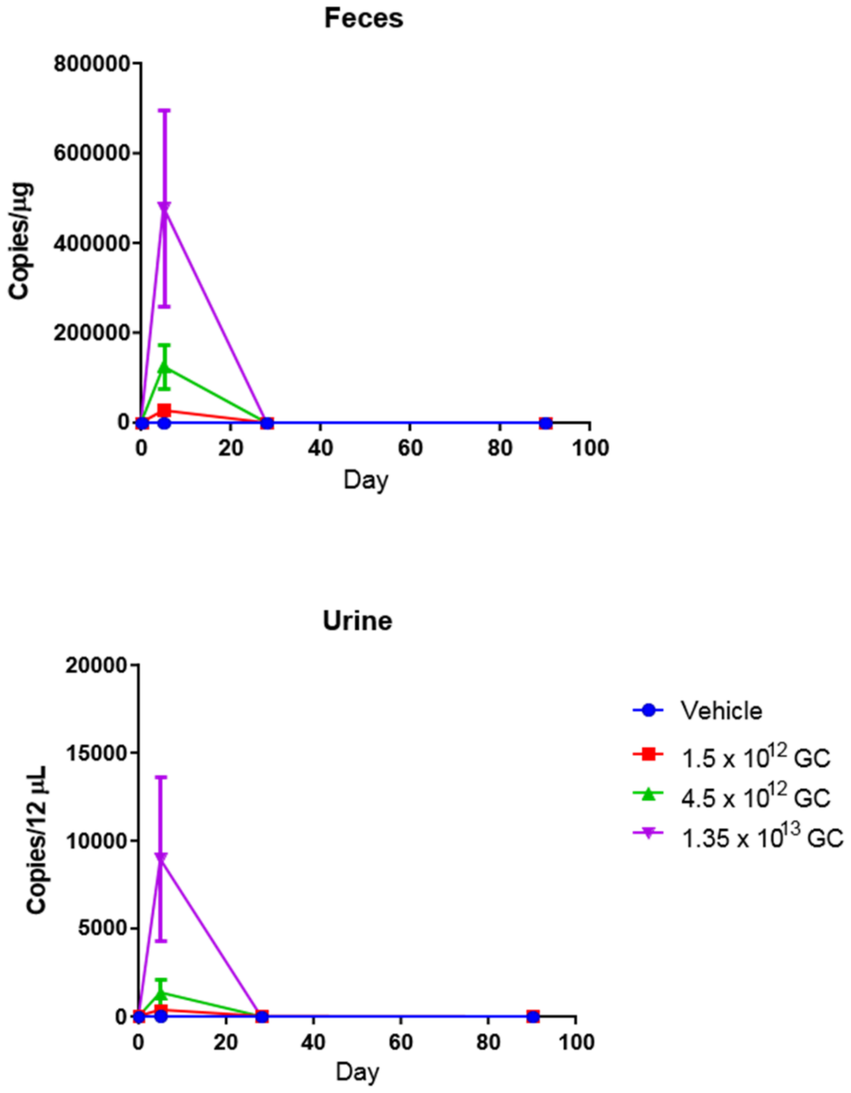

Supplemental Figure 2. Vector Excretion in Feces and Urine Following ICM Administration to NHPs

Each line represents mean vector genomes detected per µg DNA (feces) or per 12 µL of urine for each cohort. Error bars represent SEM.

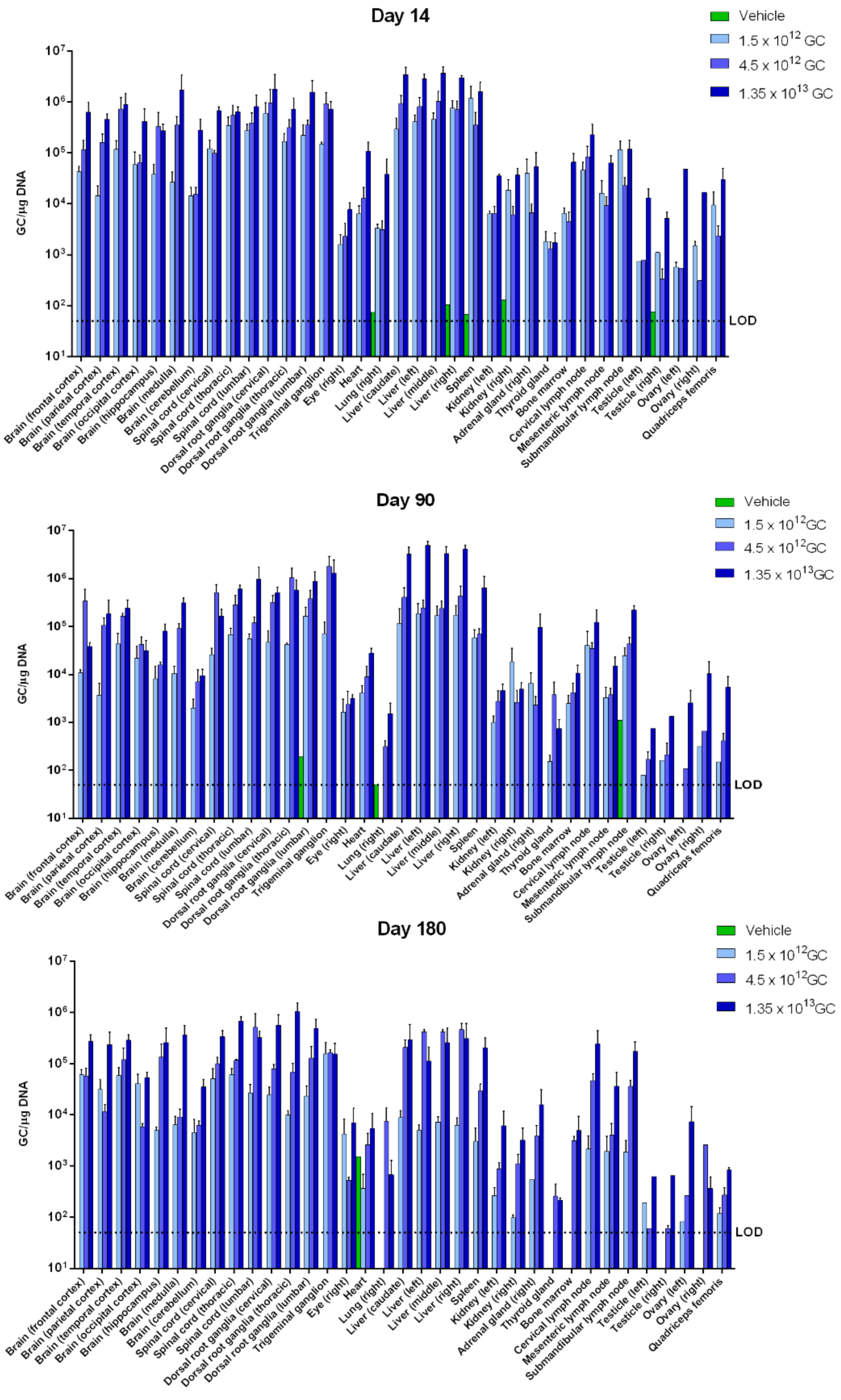

Supplemental Figure 3. Vector Biodistribution Following ICM Administration to NHPs. Each bar represents mean vector genomes detected per µg DNA. Error bars represent SEM. LOD = limit of detection (50 copies per µg DNA).

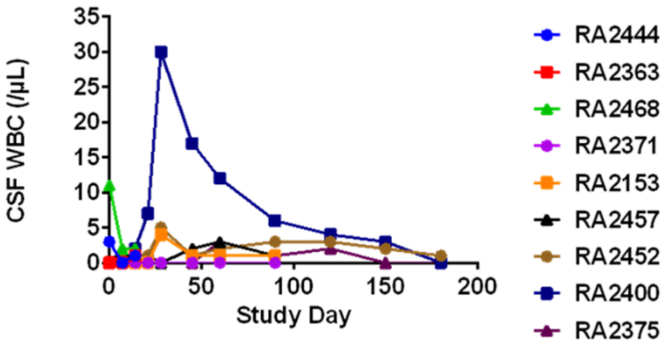

Supplemental Figure 4. CSF leukocyte counts in high dose cohort
AAVhu68 expressing human SMN was administered ICM to adult rhesus macaques at a dose of 1.35 x 10^{13} GC on Day 0. WBC = white blood cells.

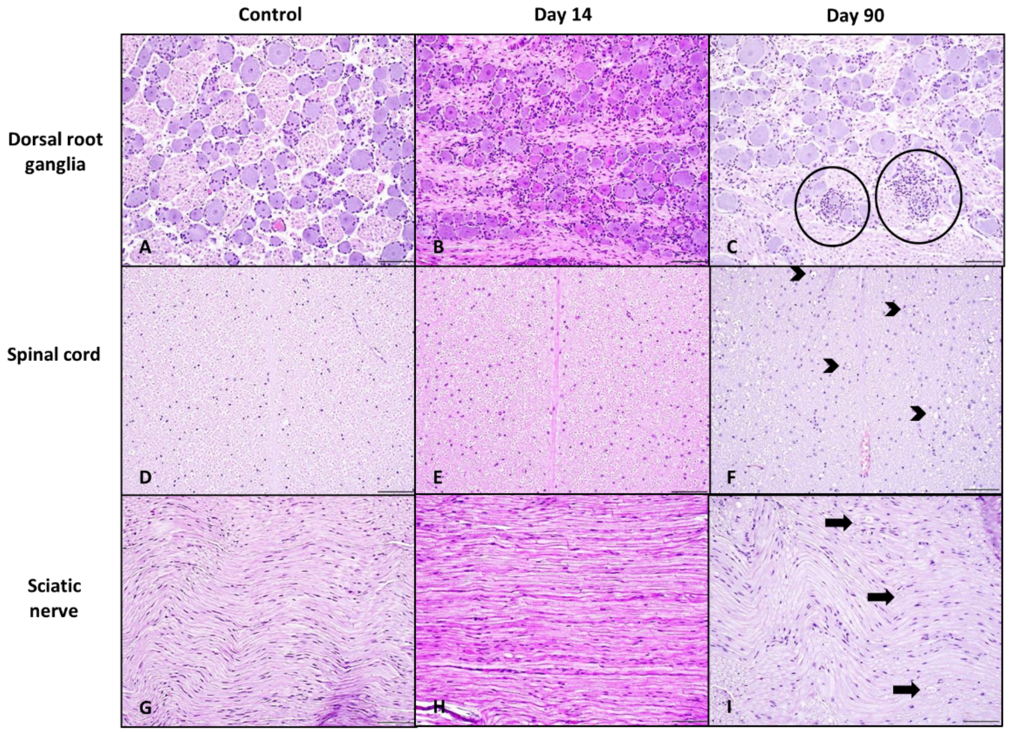

Supplemental Figure 5. Representative central and peripheral nervous system histopathologic findings in nonhuman primates administered AAVhu68 expressing human SMN via ICM injection. Pertinent vector-related findings were observed in the central and peripheral nervous system, primarily at the day 90 and day 180 time-point, with no dose effect. At the day 14 time-point, dorsal root ganglia (DRG) from a majority of vector-treated animals were histologically normal (A-B). Single neuronal degeneration was sporadically observed in few dorsal root ganglia segments and trigeminal ganglia of vector-treated animals as well as the trigeminal ganglia of the day 14 control animal; however, definitive interpretation of these findings were difficult due to limited number of control animals. No significant histologic findings were noted in the spinal cord (D-E) and peripheral nerves (shown: sciatic nerve; G-H) of vehicle control and vector-treated animals from the day 14 time-point. The dorsal root ganglia in few segments from treated groups at the day 90 time-point (C) had minimal neuronal cell body degeneration (circles) characterized by central chromatolysis, satellitosis and mononuclear cell infiltrates that surrounded and invaded neuronal cell bodies (neuronophagia). The majority of animals from all vector-treated groups had a minimal to moderate axonopathy of the dorsal white matter tracts of the spinal cord (F), which was bilateral and characterized by dilated myelin sheaths with and without myelomacrophages (arrowheads), consistent with axonal degeneration. The majority of these animals had a similar minimal to mild axonopathy (arrows) in peripheral nerves of both the fore- and hindlimbs (shown: sciatic nerves; I). A dose effect was not observed in any of the test article related findings. Findings at the day 180 time point were similar to those shown for Day 90, although severity was lower on Day 180. (Hematoxylin and eosin; Scale bar = 100 μm).

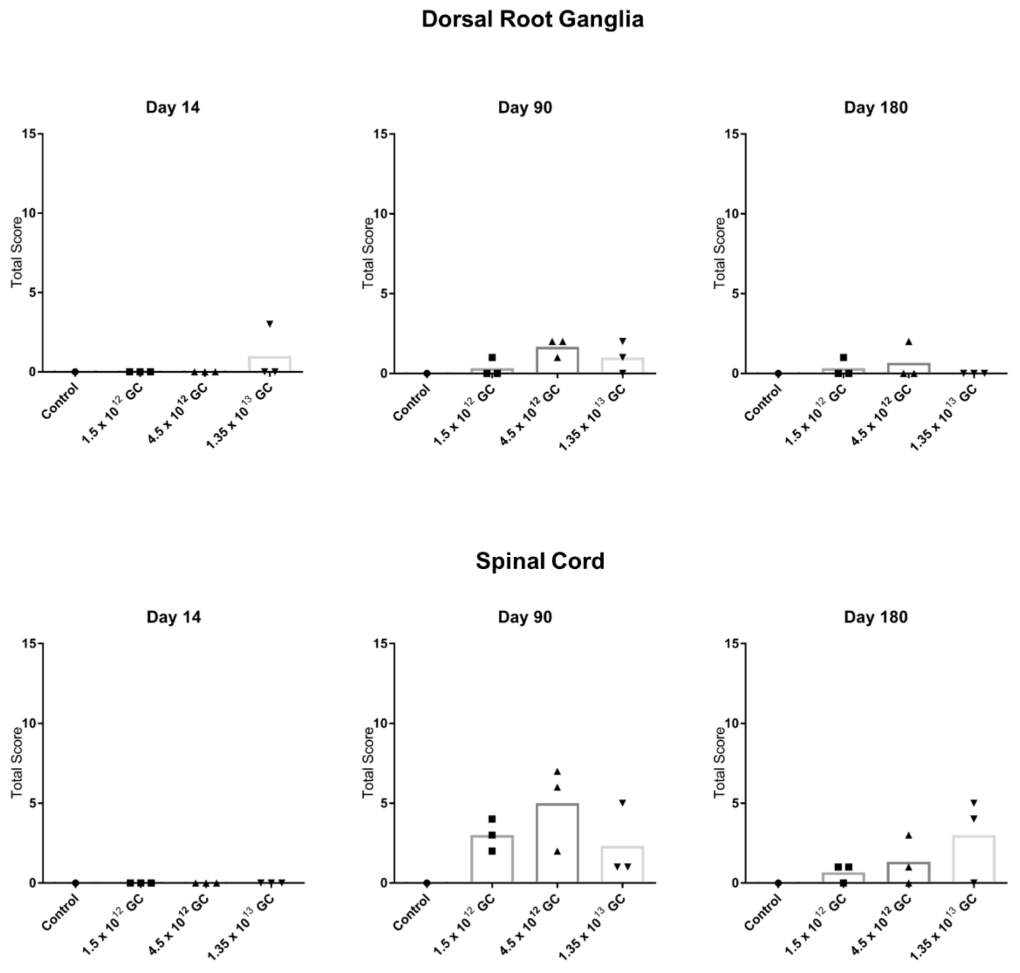

Supplemental Figure 6. Histological findings in primary sensory neurons of adult NHPs after ICM administration of AAVhu68 expressing human SMN.

A board-certified veterinary pathologist reviewed sections of dorsal root ganglia and spinal cord from the cervical, thoracic and lumbar levels. Neuronal cell body degeneration in dorsal root ganglia and axonal degeneration in the dorsal columns of the spinal cord were assigned a score of 0 (no findings present), or a severity score ranging from 1 (minimal) to 5 (severe). The total score was calculated for each animal by adding the score from the cervical, thoracic and lumbar levels. The maximum severity score possible is 15.

Dose (GC)	Animal ID	Necropsy Day	AAVhu68 NAb_{50}								
			Study Day								
			0	14	28	45	60	90	120	150	180
Vehicle	RA2424	14	5	< 5							
1.5×10^{12}	RA2031		< 5	160							
	RA1156		< 5	80							
	RA0704		< 5	160							
4.5×10^{12}	RA2463		5	20							
	RA2410		< 5	160							
	RA2433		< 5	80							
1.35×10^{13}	RA2444		< 5	160							
	RA2363		< 5	320							
	RA2468		< 5	160							
Vehicle	RA2456	90 ± 2	< 5	< 5	< 5	< 5	< 5	< 5			
1.5×10^{12}	RA2369		< 5	80	40	80	320	160			
	RA2412		5	160	160	320	640	80			
	RA0549		< 5	80	40	1280	640	320			
4.5×10^{12}	RA1875		< 5	80	80	2560	2560	2560			
	RA1853		< 5	160	320	1280	1280	2560			
	RA2360		< 5	80	160	2560	2560	320			
1.35×10^{13}	RA2371		5	40	40	1280	640	640			
	RA2153		< 5	160	320	2560	1280	1280			
	RA2457		< 5	40	160	2560	640	320			
Vehicle	RA2382	180 ± 2	< 5	< 5	< 5	< 5	< 5	< 5	< 5	< 5	< 5
1.5×10^{12}	RA2464		< 5	2560	320	1280	1280	1280	1280	1280	640
	RA2434		< 5	5	80	640	640	320	640	320	320
	RA2413		< 5	160	320	320	320	320	640	320	320
4.5×10^{12}	RA2467		< 5	160	160	640	640	320	320	320	160
	RA2426		< 5	80	640	640	640	640	1280	1280	320
	RA2150		< 5	160	80	640	1280	640	640	640	320
1.35×10^{13}	RA2452		< 5	80	40	160	640	160	320	160	320
	RA2400		20	160	1280	1280	2560	1280	1280	1280	640
	RA2375		5	160	160	640	1280	1280	1280	1280	640

Supplemental Figure 7. Neutralizing antibody responses to the vector capsid in serum of adult NHPs after ICM administration of AAVhu68 expressing human SMN.
The neutralizing antibody titer values reported are the reciprocal dilutions of serum at which in vitro cell transduction was reduced by at least 50% compared to control wells (without sample). The limit of detection was 1:5 dilution of sample. Day 0 samples were collected immediately prior to vector administration.

Dose (GC)	Necropsy Day	Animal ID	Antigen	PBMC								Liver	Spleen	Bone Marrow
				Study Day										
				0	14	28	60	90	120	150	180			
Vehicle	14	RA2424	AAVhu68	-	-							-	-	-
			hSMN	-	-							-	-	-
$1.5x10^{12}$		RA2031	AAVhu68	-	-							+	-	-
			hSMN	-	-							+	-	-
		RA1156	AAVhu68	-	-							-	-	-
			hSMN	-	-							-	-	+
		RA0704	AAVhu68	-	-							-	-	-
			hSMN	-	-							-	-	+
$4.5x10^{12}$		RA2463	AAVhu68	-	-							-	-	-
			hSMN	-	-							-	-	-
		RA2410	AAVhu68	-	-							-	-	-
			hSMN	-	-							-	-	+
		RA2433	AAVhu68	-	-							-	-	-
			hSMN	-	-							-	-	-
$1.35x10^{13}$		RA2444	AAVhu68	-	-							-	-	-
			hSMN	-	-							-	-	-
		RA2363	AAVhu68	-	-							-	-	-
			hSMN	-	-							-	-	-
		RA2468	AAVhu68	+	+							-	-	-
			hSMN	-	-							-	-	-
Vehicle	90 ± 2	RA2456	AAVhu68	-	-	-	-	-				-	-	-
			hSMN	-	-	-	-	-				-	-	-
$1.5x10^{12}$		RA2369	AAVhu68	-	-	-	-	-				-	-	-
			hSMN	-	-	-	-	+				+	+	-
		RA2412	AAVhu68	-	-	-	-	-				+	-	-
			hSMN	-	-	-	-	-				-	-	-
		RA0549	AAVhu68	-	-	+	+	-				+	-	-
			hSMN	-	-	-	+	+				+	-	+
$4.5x10^{12}$		RA1875	AAVhu68	-	-	-	-	-				+	-	+
			hSMN	-	-	+	-	-				+	-	+
		RA1853	AAVhu68	-	-	-	-	-				-	-	-
			hSMN	-	-	-	-	-				-	-	-
		RA2360	AAVhu68	+	-	+	+	+				-	-	-
			hSMN	-	-	-	-	+				+	-	-
$1.35x10^{13}$		RA2371	AAVhu68	+	+	+	+	+				+	+	-
			hSMN	-	-	-	+	+				+	+	+
		RA2153	AAVhu68	-	-	-	-	-				+	-	-
			hSMN	-	-	-	-	-				-	-	-
		RA2457	AAVhu68	-	-	-	-	-				-	-	-
			hSMN	-	-	-	+	+				+	+	+
Vehicle	180 ± 2	RA2382	AAVhu68	-	-	-	-	-	-	-	-	-	-	-
			hSMN	-	-	-	-	-	+	+	-	-	-	-
$1.5x10^{12}$		RA2464	AAVhu68	-	+	+	+	-	+	-	-	-	-	-
			hSMN	-	-	-	+	-	-	-	-	-	-	-
		RA2434	AAVhu68	-	-	-	-	-	-	-	-	-	-	-
			hSMN	-	-	-	+	+	+	-	-	+	-	+
		RA2413	AAVhu68	-	-	-	-	-	-	-	+	+	-	-
			hSMN	-	-	-	-	-	-	-	-	-	-	-
$4.5x10^{12}$		RA2467	AAVhu68	-	-	-	-	-	-	-	-	-	-	-
			hSMN	-	-	-	-	-	-	-	-	-	-	-
		RA2426	AAVhu68	-	-	-	+	+	+	+	+	+	-	-
			hSMN	-	-	-	+	+	+	+	+	+	+	-
		RA2150	AAVhu68	-	-	-	-	+	-	-	-	-	-	-
			hSMN	-	-	-	-	+	+	+	-	-	-	-
$1.35x10^{13}$		RA2452	AAVhu68	-	-	-	-	-	-	-	-	-	-	-
			hSMN	-	-	-	-	-	-	-	-	-	-	-
		RA2400	AAVhu68	-	+	-	-	-	-	-	-	-	-	-
			hSMN	-	-	+	-	-	+	-	-	-	-	-
		RA2375	AAVhu68	-	-	-	-	-	+	+	-	-	-	-
			hSMN	-	-	-	-	-	-	-	-	-	-	-

Supplemental Figure 8. IFN gamma ELISPOT detection of T cell responses to AAVhu68 capsid and hSMN transgene. Lymphocytes were isolated from peripheral blood, bone marrow, liver and spleen. T cell responses were measured against pooled overlapping 15-mer peptides comprising the AAVhu68 VP1 sequence (3 peptide pools) or the human SMN sequence (2 peptide pools). + indicates a detectable T cell response (>55 spot forming units and 3-fold greater than unstimulated control) to the indicated antigen. PBMC, peripheral blood mononuclear cells.
