# Supplementary material for: Translational Feasibility of Lumbar Puncture for Intrathecal AAV Administration
Source: Mol Ther Methods Clin Dev. 2020 Apr 18;17:969–74. doi: 10.1016/j.omtm.2020.04.012 (PMC7218226; doi:10.1016/j.omtm.2020.04.012)
Supplement: Document S2. Article plus Supplemental Information [file mmc2.pdf]

Molecular Therapy
Methods & Clinical Development
Original Article

Translational Feasibility of Lumbar Puncture for Intrathecal AAV Administration

Christian Hinderer,[1] Nathan Katz,[1] Cecilia Dyer,[1] Tamara Goode,[1] Julia Johansson,[1] Peter Bell,[1] Laura Richman,[1] Elizabeth Buza,[1] and James M. Wilson[1]

[1]Gene Therapy Program, Perelman School of Medicine, University of Pennsylvania, Philadelphia, PA, USA

Preclinical studies have demonstrated that a single injection of an adeno-associated virus (AAV) vector into the cerebrospinal fluid (CSF) can achieve widespread gene transfer throughout the central nervous system. Successfully translating this approach to humans requires identifying factors that influence AAV distribution in the CSF so that optimal parameters can be replicated in the clinic. In the context of developing a motor neuron-targeted gene therapy for spinal muscular atrophy, we conducted studies in nonhuman primates to evaluate the impact of injection volume on spinal cord transduction after AAV delivery via lumbar puncture. Lumbar injection of an AAVhu68 vector targeted motor neurons throughout the spinal cord, but only in juvenile nonhuman primates administered large injection volumes, equivalent to about half of the total CSF volume. Upon repeating this study with clinically relevant injection volumes and larger animals, we found that lumbar puncture failed to achieve significant transduction of the spinal cord. In contrast, vector administered into the cisterna magna distributed reproducibly throughout the spinal cord in both juvenile and adult animals. These findings highlight the challenges of translating AAV delivery via lumbar puncture to humans and suggest that delivery into the cisterna magna may represent a more feasible alternative.

INTRODUCTION

Adeno-associated virus (AAV)-mediated gene transfer has demonstrated the potential for long-term expression of a transgene in the human brain with an acceptable safety profile.[1–3] Most evidence of persistent gene transfer has been limited to clinical trials in which the vector was injected directly into the brain parenchyma.[1–3] Although this approach is promising for some diseases, intraparenchymal injection is an invasive procedure that results in limited distribution of the vector beyond the injection site, thus making it unsuitable for many applications.[3,4] Expanding the potential of AAV gene therapy to new targets will require the identification of capsids and delivery methods that can broadly target cells relevant to each disease. Intravenous AAV delivery offers a noninvasive alternative capable of broad vector distribution. When injected intravenously at high doses, AAV serotype 9 (AAV9) can efficiently transduce primary sensory neurons in the dorsal root ganglia and lower motor neurons in the spinal cord and brainstem.[5] The latter finding led to the development of Zolgensma (onasemnogene abeparvovec-xioi), an intravenous AAV9 gene therapy for the motor neuron disease spinal muscular atrophy (SMA).[6] Although delivering AAV9 intravenously can efficiently target motor and sensory neurons that project to the periphery, brain transduction is comparatively modest with this approach.[7,8] Clinical applications of intravenous delivery are limited by pre-existing AAV neutralizing antibodies, manufacturing challenges that arise from the large required doses, and systemic toxicity associated with these high doses.[5] AAV delivery into the cerebrospinal fluid (CSF) has several advantages: (1) it requires much lower vector doses, (2) it is unaffected by serum neutralizing antibodies,[9–11] and (3) it can achieve broad transduction in the brain and spinal cord with a single injection.[8–10] Transduction in the brain is diffuse, with only a small percentage of cells expressing the transgene; however, certain cell populations such as the lower motor neurons in the spinal cord are transduced at high frequencies.[8]

In large-animal studies, transduction of the lower motor neurons has been demonstrated after AAV delivery into the CSF via the lateral cerebral ventricles, cisterna magna, or lumbar cistern.[8,12] Injection via lumbar puncture has clear advantages given that this is a widely used approach in clinical practice. However, we and others have previously demonstrated that in contrast with intra-cisterna magna (ICM) injection, lumbar puncture results in a dramatically lower vector distribution to the brain and cervical spinal cord of nonhuman primates (NHPs).[8,13,14] Others have also observed limited distribution of AAV from a lumbar puncture in pigs.[15] In contrast, Meyer et al.[16] demonstrated efficient transduction of the brain and spinal cord after AAV administration to NHPs through a lumbar puncture. The authors showed that transduction could be further increased by placing animals in the Trendelenburg position, but even without this maneuver, transduction was significantly higher than others have observed following lumbar delivery. Meyer et al.[16] utilized animals that were substantially smaller and younger than those we employed in our studies, and also mixed the vector with contrast material, resulting in a different formulation and a larger injection volume. We, therefore, explored how these variables impact distribution of

Received 3 February 2020; accepted 13 April 2020;
https://doi.org/10.1016/j.omtm.2020.04.012.

Correspondence: James M. Wilson, Gene Therapy Program, Perelman School of Medicine, University of Pennsylvania, 125 S. 31st Street, Suite 1200 TRL, Philadelphia, PA 19104-3403, USA.
E-mail: wilsonjm@upenn.edu

This is an open access article under the CC BY license (http://creativecommons.org/licenses/by/4.0/).

Table 1. Study Design to Compare AAVhu68 Administration via Intracisterna Magna (ICM) Injection or Lumbar Puncture (LP) in Adult and Juvenile Rhesus Macaques

Age	Route of Administration	Dose Volume	Number of Animals
Adult (3–6 years)	ICM	1 mL	3
	LP	5 mL	4
Juvenile (12–14 months)	ICM	1 mL	3
	LP	5 mL	2

Macaques were administered 3×10^{13} GC of an AAVhu68 vector expressing a human SMN transgene. The study duration was 28 days.

an AAV vector from a lumbar puncture to determine whether changes to the procedure could allow a lumbar intrathecal approach to be translated to humans.

RESULTS

We first attempted to replicate the prior report[16] of efficient motor neuron transduction in juvenile animals by using a high-volume lumbar AAV injection. In this previous study, the administered injection was a vector mixed with contrast material, which resulted in a higher volume and an increased density.[16] We tested whether a large injection volume without contrast could replicate these findings. Juvenile (12- to 14-month-old) rhesus macaques were administered 3×10^{13} genome copies (GC) of an AAVhu68 vector expressing a human survival of motor neuron (SMN) transgene either via lumbar puncture (5 mL volume) or ICM injection (1 mL volume; see Table 1). *In situ* hybridization (ISH) for the transgene mRNA revealed extensive transduction of motor neurons throughout the spinal cord in both the lumbar puncture and ICM groups (Figure 1). In contrast, when we performed the same comparison in adult animals, only ICM delivery resulted in significant motor neuron transduction.

Widespread motor neuron transduction after lumbar AAV injection in juvenile, but not adult, NHPs could be explained by mechanical factors, with the large injection volume coupled with the animal's small size and CSF volume driving cranial diffusion of the bolus. Alternatively, this finding could be explained by intrinsic biological differences between juvenile and adult animals, such as developmental or anatomic differences that alter vector exposure to target cells. We evaluated these possibilities by administering an AAVhu68 vector via lumbar puncture to animals ranging in age from juvenile (12–14 months) to adult (3 years). A control group was composed of 3-year-old macaques that were treated by ICM injection (Table 2). To determine whether the transduction after lumbar administration was solely due to the large volume administered, we selected a clinically relevant injection volume for this study. We estimated a pediatric intrathecal dosing volume of 5 mL in patients with a total CSF volume of about 100 mL. We then scaled the dose volume to the 13 mL CSF volume of an adult rhesus macaque, resulting in a dose volume of 0.7 mL. Vector administration via lumbar puncture in this volume resulted in minimal spinal cord transduction in all animals, regardless of age (Figure 2). By contrast, ICM injection in adult animals using the same dose and volume transduced an average of 20% of motor neurons at the cervical level and 50% at the lumbar level.

We conducted good laboratory practices (GLP)-compliant toxicology studies in both adult and juvenile rhesus macaques using an AAVhu68 vector expressing the human SMN transgene. The study in juvenile animals employed lumbar intrathecal delivery with a dose volume of 2.5 mL and vector doses of 4.5×10^{12} and 1.35×10^{13} GC. We euthanized animals 1, 3, or 6 months after vector administration and quantified motor neuron transduction using ISH (Figure 3). Transduction was apparent at all time points, with more than 10% of motor neurons transduced in the lumbar segment of some animals. Overall transduction was at least 10-fold lower than observed in the prior juvenile study at a similar time point, potentially due to the lower vector dose and injection volume. The low transduction achieved in this study precluded meaningful analysis of safety.

The toxicology study performed in adult rhesus macaques employed ICM administration and a 1 mL injection volume (Table S1). Transduction of motor neurons was apparent at all necropsy time points, ranging from 14 to 180 days after vector administration (Figure 4). Motor neuron transduction was lower than observed in the prior adult NHP study in which the same vector was administered via an ICM injection at a dose that was roughly 2-fold higher. About 20% of lumbar and thoracic motor neurons were transduced with less transduction in the cervical spinal cord. A dose-response was not clear, and trends were difficult to evaluate due to the modest overall transduction levels. Transgene expression persisted throughout the 6-month study.

Vector DNA was detectable in CSF and peripheral blood; there was a correlation between peak concentrations in CSF and dose (Figure S1). The concentration of vector DNA in CSF rapidly declined following the first evaluated time point (day 7). Vector genomes in blood declined more slowly, which may be attributable to transduction of peripheral blood cells. At 180 days after administration, vector genomes were undetectable in the blood of all animals and were detectable at low levels (51 copies/12 μL) in the CSF of only one animal. Vector genomes were detectable in the urine and feces 5 days after administration; there was a correlation between peak concentrations and dose (Figure S2). Excretion rapidly declined, with most animals exhibiting undetectable levels in both urine and feces by day 28. On day 90, all animals except one had reached undetectable levels of vector DNA in urine. Only one animal in the high-dose cohort had detectable vector DNA in feces (60 copies/μg DNA) on day 90, which was near the limit of detection (50 copies/μg DNA).

Using quantitative PCR, we detected vector genomes at high levels in the brain, spinal cord, dorsal root ganglia, liver, and spleen (see Figure S3), consistent with previous studies of ICM AAV administration.[10] Lower levels of vector DNA were detectable in most tissues sampled, including skeletal muscle, lung, kidney, lymph nodes, and gonads. The quantity of vector genomes detected in CNS tissues was generally dose dependent. Animals euthanized 90 and 180 days

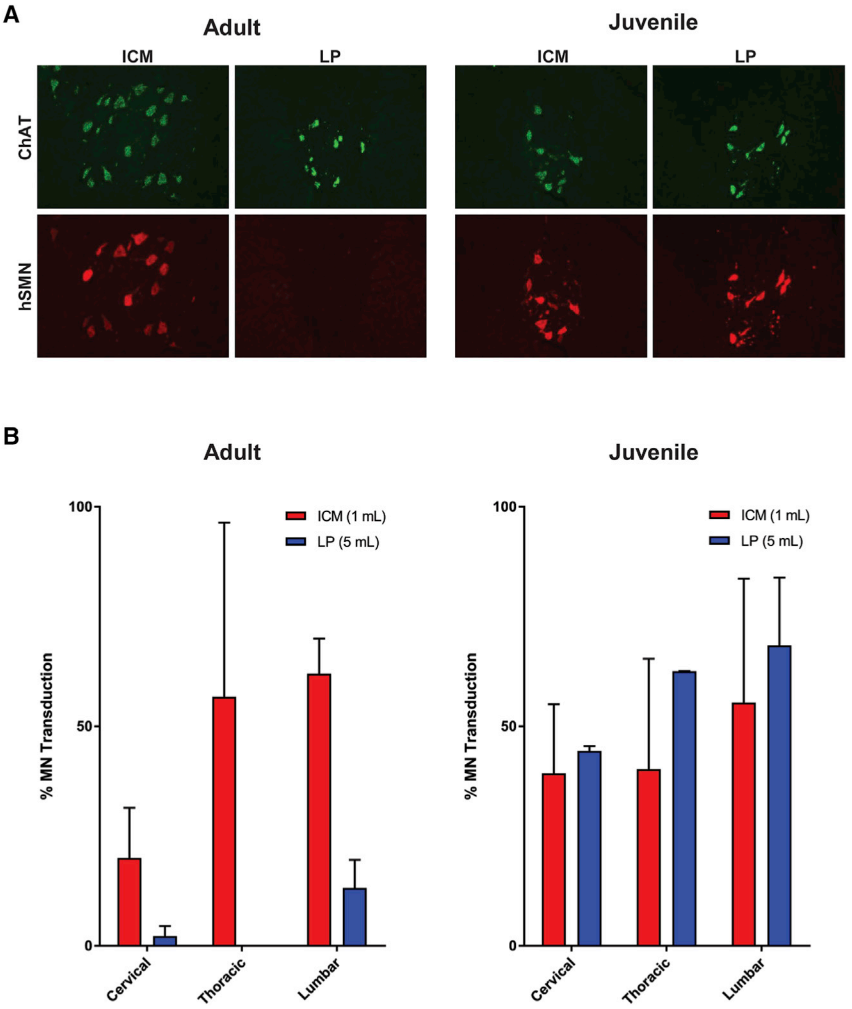

Figure 1. Motor Neuron Transduction following AAVhu68 Administration via Intra-cisterna Magna (ICM) Injection or Lumbar Puncture (LP) to Adult and Juvenile NHPs

Adult (3- to 6-year-old) and juvenile (12- to 14-month-old) rhesus macaques were administered 3 $\times$ 10^{13} GC of an AAVhu68 vector expressing human SMN from a chicken beta-actin promoter. The vector was injected into the cisterna magna (ICM) in a volume of 1 mL or via LP in a volume of 5 mL. Animals were necropsied 28 days after vector administration. We performed *in situ* hybridization on spinal cord sections using probes for the human SMN transgene and choline acetyltransferase (A). We quantified the percentage of $ChAT^+$ motor neurons expressing human SMN in spinal cord sections from cervical, thoracic, and lumbar levels (B). Error bars = standard error of the mean (SEM).

after vector administration exhibited modestly lower vector genome levels in tissues compared with animals euthanized on day 14. This indicates that some vector genomes detected soon after administration do not represent stable transduction. Vector genomes in CNS tissues appeared stable between 90 and 180 days after injection.

Table 2. Study Design for Evaluating AAVhu68 Administration via Lumbar Puncture to NHPs of Different Ages Using a Clinically Relevant Injection Volume

Age (years)	Route of Administration	Number of Animals
3	ICM	3
1	LP	4
1.5	LP	4
2	LP	3
3	LP	3

NHPs were administered 1.5 $\times$ 10^{13} GC of an AAVhu68 vector expressing a GFP transgene at a dose of 0.7 mL. The study duration was 21 days.

We did not detect any dose-limiting toxicity in this study. One animal unexpectedly extended its neck during the ICM injection procedure, resulting in suspected needle penetration of the brainstem. Upon recovery from anesthesia, this animal exhibited mild unilateral weakness. The animal was treated with a short course of prednisone, and the motor deficit resolved within a week of the injection. There were no other clinical abnormalities noted throughout the study. None of the animals exhibited any clinically meaningful changes in hematology, coagulation, or blood and CSF clinical chemistry parameters. One animal treated with the highest vector dose exhibited an asymptomatic self-limited lymphocytic pleocytosis between 28 and 60 days after vector administration (Figure S4).

Similar to previous studies of intrathecal AAV administration, all groups exhibited asymptomatic minimal degeneration of dorsal root ganglia sensory neurons and minimal-to-moderate degeneration of their associated axons in the dorsal white matter tracts of the spinal cord with no clear dose dependence (Figures S5 and S6).[10] These findings emerged on day 14 and developed fully by day 90, with no further progression from days 90 to 180.

Neutralizing antibodies to the AAVhu68 capsid were elicited in all vector-treated animals (Figure S7). Interferon-gamma T cell responses to the capsid and transgene product were detectable in peripheral blood, as well as in lymphocytes harvested from the liver, spleen, and bone marrow of some animals (Figure S8). T cell responses to the AAVhu68 capsid occurred in 12 of 27 vector-treated animals, and 15 of 27 exhibited responses to the human SMN protein, with similar frequencies in all dose cohorts. T cell responses were not associated with abnormal clinical or histological findings. T cell responses to the AAVhu68 capsid were detectable prior to treatment in three animals, potentially due to prior infections with other AAV serotypes with conserved T cell epitopes.

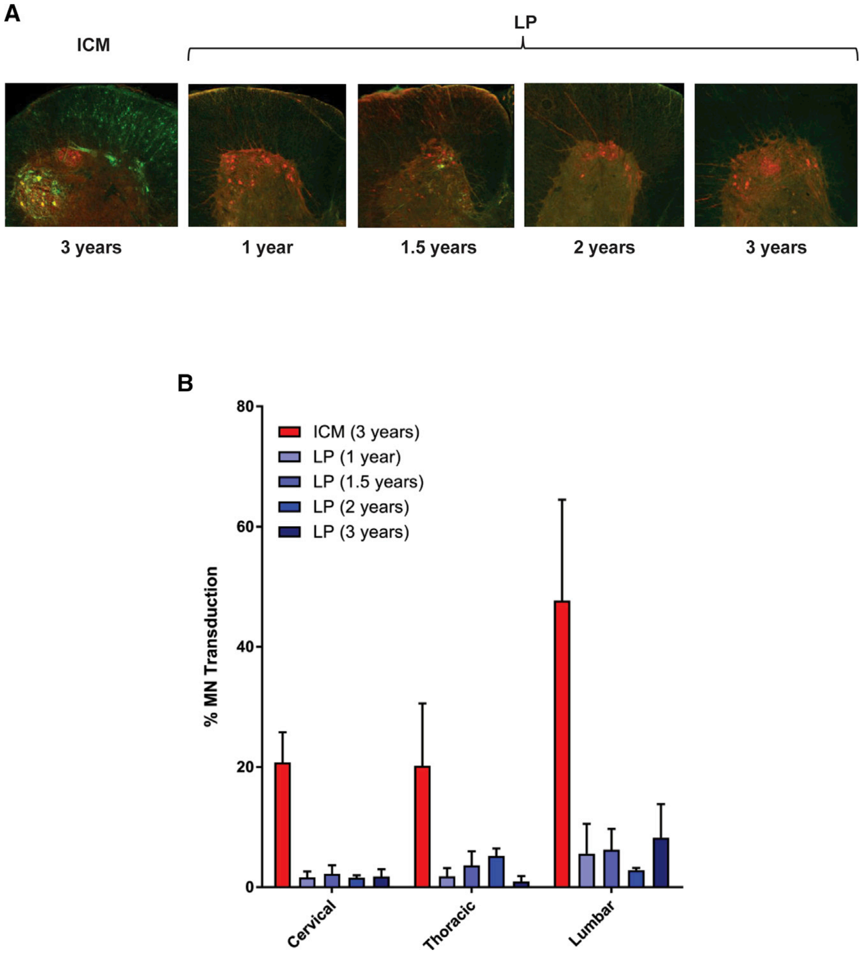

Figure 2. Motor Neuron Transduction following ICM or LP AAVhu68 Administration to NHPs of Different Ages Using Clinically Relevant Injection Volumes

Rhesus macaques ranging in age from 1 to 3 years were administered 1.5 × 10^{13} GC of an AAVhu68 vector expressing GFP via LP in a volume of 0.7 mL. A control group of 3-year-old NHPs was administered the same vector and dose volume via ICM injection. Animals were necropsied 21 days after vector administration. We performed immunostaining on spinal cord sections using antibodies against ChAT (red) and GFP (green) (A). We quantified the percentage of $ChAT^+$ motor neurons expressing GFP in spinal cord sections from cervical, thoracic, and lumbar levels (B). Error bars = SEM.

DISCUSSION

The present studies elucidate key determinants of AAV distribution in CSF and help explain inconsistencies in previous studies. The results of ICM vector administration in NHPs were consistent with our prior studies[8,10,17] and other reports from the literature.[18] Although we previously reported that lumbar puncture is far less effective than ICM AAV administration in NHPs,[8] here we were able to reproduce the successful gene transfer to the spinal cord reported by another group by more closely replicating aspects of their model system and injection method.

Following ICM AAV administration, we consistently observed a lumbar-to-cervical gradient of motor neuron transduction despite the greater distance from the lumbar region to the injection site. We hypothesize that this is related to the anatomy of the lumbar motor neurons, the axons of which may be more exposed to CSF due to the long ventral roots of the lumbar spinal cord. In the present studies, we observed a nonlinear dose-response with markedly lower motor neuron transduction when the dose of the human SMN-expressing vector was decreased from 3 × 10^{13} GC in pilot studies to 1.35 × 10^{13} GC in the toxicology study. This observation may have been partly related to the low sensitivity of ISH for detecting transgene expression, as the study conducted with a GFP reporter gene demonstrated higher overall transduction levels. However, our results suggest that relatively high doses, equivalent to an NHP dose of at least 1.5 × 10^{13} GC, may be required to achieve reliable motor neuron transduction, particularly of the cervical spinal cord. ICM vector administration had a similar safety profile to previous studies;[10,17] mild transient pleocytosis and minimal asymptomatic degeneration of sensory neurons in the dorsal root ganglia were the only vector-related adverse effects.

Our findings suggest that AAV delivery via lumbar puncture, although feasible in nonclinical studies, will be challenging to translate to humans. Delivery to the entire spinal cord was possible only when the vector was administered in a large volume in small NHPs, indicating that the distribution of the vector was driven by the spread of the injected bolus rather than by circulation of the vector in CSF. Directly replicating this approach in humans may require administering roughly half of the total CSF volume, which is inconsistent with clinical practice and may cause toxicity from rapid changes in intracranial pressure and CSF electrolytes. Moreover, others have demonstrated that cranial spread of a lumbar bolus is impaired if the dura has been punctured in a previous injection attempt.[13] If administering a one-time therapy relies on a lumbar puncture that is always successful on the first attempt, then an unacceptable level of variability may be apparent in clinical outcomes.

These studies were carried out in the context of developing a gene therapy for SMA. Our analysis was therefore focused on the transduction of motor neurons in the spinal cord, although we expect these findings to be broadly applicable to programs that target the spinal cord or brain. The challenges of AAV distribution to the spinal cord following lumbar injection are likely to be further

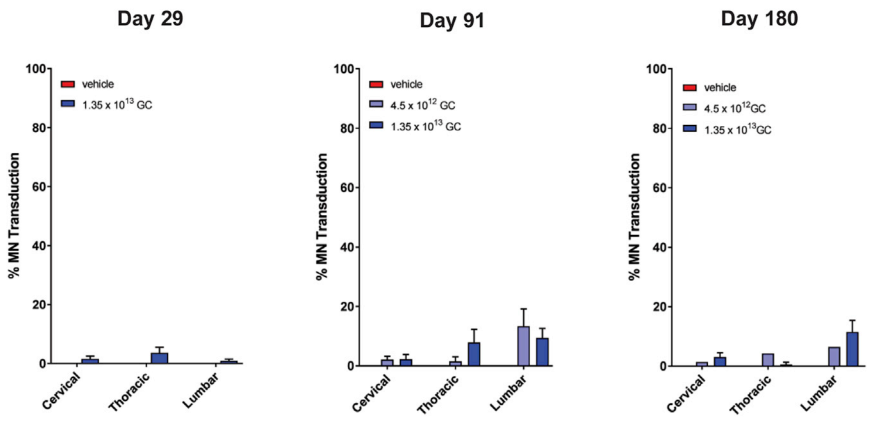

Figure 3. Motor Neuron Transduction in Juvenile NHP Toxicology Study Utilizing LP Injection

Juvenile (12- to 14-month-old) rhesus macaques were administered 1.35×10^{13} or 4.5×10^{12} GC of an AAVhu68 vector expressing human SMN in a volume of 2.5 mL via LP. Control animals were treated with vehicle. Animals were necropsied 29, 91, or 180 days after vector administration. We quantified the percentage of $ChAT^+$ motor neurons expressing human SMN in spinal cord sections from cervical, thoracic, and lumbar levels using ISH. Error bars = SEM.

amplified when the brain is the primary target. Our findings suggest that ICM administration may overcome these delivery challenges.

MATERIALS AND METHODS

Vectors

Vectors were produced by triple transfection of adherent HEK293 cells and purified by ultracentrifugation on an iodixanol gradient (GFP vectors) or by affinity chromatography (SMN vectors) as previously described.[5]

Animal Procedures

All animal protocols were approved by the Institutional Animal Care and Use Committee of the University of Pennsylvania. Adult NHPs were 3–8 kg at the time of dosing. One-year-old NHPs were 1.5–3 kg at the time of dosing. NHPs were anesthetized with ketamine and dexmedetomidine for all procedures. ICM injection was performed as previously described.[19] Lumbar puncture was performed under fluoroscopic guidance in anesthetized animals. After inserting a spinal needle into the L4–5 or L5–6 space, we confirmed placement by CSF return and/or by injecting up to 1 mL of contrast material (Iohexol 180). After confirming placement, we injected the vector solution by hand at a rate of approximately 2 mL/min. Complete blood counts and serum chemistry panels were evaluated prior to vector administration, then weekly for 1 month and monthly thereafter. CSF (1 mL) was collected by suboccipital puncture. We performed euthanasia with a pentobarbital overdose.

Vector Biodistribution and Pharmacokinetics

We quantified the vector genomes in the blood, CSF, urine, feces, and tissue samples by TaqMan PCR as previously described.[10,17]

Immunological Assays

Neutralizing antibody assays and ELISPOTs were performed as previously described.[5]

Histology

We performed tissue fixation and ISH as previously described.[5] For quantification of motor neuron transduction, two sections from the cervical, thoracic, and lumbar levels were analyzed. The total number of choline acetyltransferase $(ChAT)^+$ motor neurons and the number of human $SMN^+/ChAT^+$ cells was determined for each spinal cord level, and mean percent transduction was calculated. For histopathology, we sectioned and stained formalin-fixed, paraffin-embedded tissues with hematoxylin and eosin according to standard protocols. A

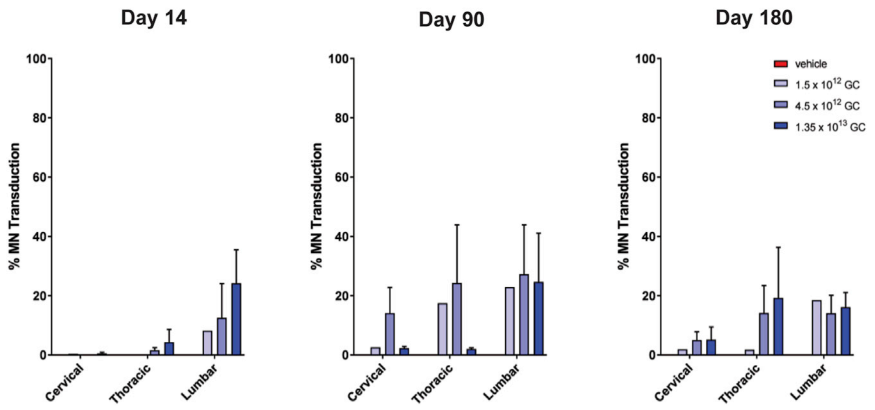

Figure 4. Motor Neuron Transduction in an Adult NHP Toxicology Study Utilizing ICM Injection

Adult rhesus macaques were administered 1.35×10^{13}, 4.5×10^{12}, or 1.5×10^{12} GC (n = 9 per dose) of an AAVhu68 vector expressing human SMN in a volume of 1 mL via ICM injection. Control animals (n = 3) were treated with vehicle. Animals were necropsied 14, 90, or 180 days after vector administration. We quantified the percentage of $ChAT^+$ motor neurons expressing human SMN in spinal cord sections from cervical, thoracic, and lumbar levels using ISH. Error bars = SEM.

board-certified veterinary pathologist examined the slides. A second pathologist peer reviewed the slides for the toxicology studies.

SUPPLEMENTAL INFORMATION

Supplemental Information can be found online at https://doi.org/10.1016/j.omtm.2020.04.012.

AUTHOR CONTRIBUTIONS

C.H., N.K., and J.M.W. designed experiments; C.H., N.K., C.D., T.G., J.J., P.B., L.R., and E.B. conducted experiments; C.H. and J.M.W. wrote the paper.

CONFLICTS OF INTEREST

J.M.W. is a paid advisor to and holds equity in Scout Bio and Passage Bio; he holds equity in Surmount Bio; he also has a sponsored research agreement with Ultragenyx, Biogen, Janssen, Precision Biosciences, Moderna Inc., Scout Bio, Passage Bio, Amicus Therapeutics, and Surmount Bio, which are licensees of Penn technology. J.M.W. is an inventor on patents that have been licensed to various biopharmaceutical companies and for which he may receive payments. C.H. is an inventor on patents licensed to biopharmaceutical companies and holds equity in Scout Bio.

ACKNOWLEDGMENTS

We would like to thank Erin Bote, Amber Hamilton, Peter Hayashi, Richard Fetterly, Barbara Rice, C. Angelica Medina-Jaszek, Mohamad Nayal, and Yanqing Zhu for technical assistance. We acknowledge the support of the Program for Comparative Medicine, Immunology Core, Vector Core, and Pathology Core of the Gene Therapy Program, University of Pennsylvania. This work was supported by a grant from Biogen.

REFERENCES

1. Mittermeyer, G., Christine, C.W., Rosenbluth, K.H., Baker, S.L., Starr, P., Larson, P., Kaplan, P.L., Forsayeth, J., Aminoff, M.J., and Bankiewicz, K.S. (2012). Long-term evaluation of a phase 1 study of AADC gene therapy for Parkinson's disease. Hum. Gene Ther. *23*, 377–381.
2. Tardieu, M., Zérah, M., Gougeon, M.L., Ausseil, J., de Bournonville, S., Husson, B., Zafeiriou, D., Parenti, G., Bourget, P., Poirier, B., et al. (2017). Intracerebral gene therapy in children with mucopolysaccharidosis type IIIB syndrome: an uncontrolled phase 1/2 clinical trial. Lancet Neurol. *16*, 712–720.
3. Kojima, K., Nakajima, T., Taga, N., Miyauchi, A., Kato, M., Matsumoto, A., Ikeda, T., Nakamura, K., Kubota, T., Mizukami, H., et al. (2019). Gene therapy improves motor and mental function of aromatic l-amino acid decarboxylase deficiency. Brain *142*, 322–333.
4. Vite, C.H., McGowan, J.C., Niogi, S.N., Passini, M.A., Drobatz, K.J., Haskins, M.E., and Wolfe, J.H. (2005). Effective gene therapy for an inherited CNS disease in a large animal model. Ann. Neurol. *57*, 355–364.
5. Hinderer, C., Katz, N., Buza, E.L., Dyer, C., Goode, T., Bell, P., Richman, L.K., and Wilson, J.M. (2018). Severe Toxicity in Nonhuman Primates and Piglets Following High-Dose Intravenous Administration of an Adeno-Associated Virus Vector Expressing Human SMN. Hum. Gene Ther. *29*, 285–298.
6. Mendell, J.R., Al-Zaidy, S., Shell, R., Arnold, W.D., Rodino-Klapac, L.R., Prior, T.W., Lowes, L., Alfano, L., Berry, K., Church, K., et al. (2017). Single-Dose Gene-Replacement Therapy for Spinal Muscular Atrophy. N. Engl. J. Med. *377*, 1713–1722.
7. Gurda, B.L., De Guilhem De Lataillade, A., Bell, P., Zhu, Y., Yu, H., Wang, P., Bagel, J., Vite, C.H., Sikora, T., Hinderer, C., et al. (2016). Evaluation of AAV-mediated gene therapy for central nervous system disease in canine mucopolysaccharidosis VII. Mol. Ther. *24*, 206–216.
8. Hinderer, C., Bell, P., Vite, C.H., Louboutin, J.P., Grant, R., Bote, E., Yu, H., Pukenas, B., Hurst, R., and Wilson, J.M. (2014). Widespread gene transfer in the central nervous system of cynomolgus macaques following delivery of AAV9 into the cisterna magna. Mol. Ther. Methods Clin. Dev. *1*, 14051.
9. Hinderer, C., Bell, P., Louboutin, J.P., Zhu, Y., Yu, H., Lin, G., Choa, R., Gurda, B.L., Bagel, J., O'Donnell, P., et al. (2015). Neonatal systemic AAV induces tolerance to CNS gene therapy in MPS I dogs and nonhuman primates. Mol. Ther. *23*, 1298–1307.
10. Hordeaux, J., Hinderer, C., Goode, T., Buza, E.L., Bell, P., Calcedo, R., Richman, L.K., and Wilson, J.M. (2018). Toxicology Study of Intra-Cisterna Magna Adeno-Associated Virus 9 Expressing Iduronate-2-Sulfatase in Rhesus Macaques. Mol. Ther. Methods Clin. Dev. *10*, 68–78.
11. Haurigot, V., Marcó, S., Ribera, A., Garcia, M., Ruzo, A., Villacampa, P., Ayuso, E., Añor, S., Andaluz, A., Pineda, M., et al. (2013). Whole body correction of mucopolysaccharidosis IIIA by intracerebrospinal fluid gene therapy. J. Clin. Invest. *123*, 3254–3271.
12. Hinderer, C., Bell, P., Katz, N., Vite, C.H., Louboutin, J.P., Bote, E., Yu, H., Zhu, Y., Casal, M.L., Bagel, J., et al. (2018). Evaluation of Intrathecal Routes of Administration for Adeno-Associated Viral Vectors in Large Animals. Hum. Gene Ther. *29*, 15–24.
13. Ohno, K., Samaranch, L., Hadaczek, P., Bringas, J.R., Allen, P.C., Sudhakar, V., Stockinger, D.E., Snieckus, C., Campagna, M.V., San Sebastian, W., et al. (2018). Kinetics and MR-based monitoring of AAV9 vector delivery into cerebrospinal fluid of nonhuman primates. Mol. Ther. Methods Clin. Dev. *13*, 47–54.
14. Taghian, T., Marosfoi, M.G., Puri, A.S., Cataltepe, O.I., King, R.M., Diffie, E.B., Maguire, A.S., Martin, D.R., Fernau, D., Batista, A.R., Kuchel, T., et al. (2020). A safe and reliable technique for CNS delivery of AAV vectors in the cisterna magna. Mol. Ther. *28*, 411–421.
15. Federici, T., Taub, J.S., Baum, G.R., Gray, S.J., Grieger, J.C., Matthews, K.A., Handy, C.R., Passini, M.A., Samulski, R.J., and Boulis, N.M. (2012). Robust spinal motor neuron transduction following intrathecal delivery of AAV9 in pigs. Gene Ther. *19*, 852–859.
16. Meyer, K., Ferraiuolo, L., Schmelzer, L., Braun, L., McGovern, V., Likhite, S., Michels, O., Govoni, A., Fitzgerald, J., Morales, P., Foust, K.D., et al. (2015). Improving Single Injection CSF Delivery of AAV9-mediated Gene Therapy for SMA: A Dose-response Study in Mice and Nonhuman Primates. Mol. Ther. *23*, 477–487.
17. Hordeaux, J., Hinderer, C., Goode, T., Katz, N., Buza, E.L., Bell, P., Calcedo, R., Richman, L.K., and Wilson, J.M. (2018). Toxicology Study of Intra-Cisterna Magna Adeno-Associated Virus 9 Expressing Human Alpha-L-Iduronidase in Rhesus Macaques. Mol. Ther. Methods Clin. Dev. *10*, 79–88.
18. Samaranch, L., Salegio, E.A., San Sebastian, W., Kells, A.P., Bringas, J.R., Forsayeth, J., and Bankiewicz, K.S. (2013). Strong cortical and spinal cord transduction after AAV7 and AAV9 delivery into the cerebrospinal fluid of nonhuman primates. Hum. Gene Ther. *24*, 526–532.
19. Katz, N., Goode, T., Hinderer, C., Hordeaux, J., and Wilson, J.M. (2018). Standardized Method for Intra-Cisterna Magna Delivery Under Fluoroscopic Guidance in Nonhuman Primates. Hum. Gene Ther. Methods *29*, 212–219.

OMTM, Volume *17*

Supplemental Information

Translational Feasibility of Lumbar Puncture

for Intrathecal AAV Administration

Christian Hinderer, Nathan Katz, Cecilia Dyer, Tamara Goode, Julia Johansson, Peter Bell, Laura Richman, Elizabeth Buza, and James M. Wilson

Treatment (Dose)	Animal ID	Sex	Necropsy (days post injection)
Vehicle (N/A)	RA2424	Male	14
	RA2456	Male	90±2
	RA2382	Female	180±2
Low dose (1.50 x 10^{12} GC)	RA1156	Male	14
	RA2031	Female	
	RA0704	Female	
	RA0549	Male	90±2
	RA2369	Female	
	RA2412	Female	
	RA2464	Male	180±2
	RA2434	Male	
	RA2413	Female	
Mid-dose (4.50 x 10^{12} GC)	RA2463	Male	14
	RA2433	Male	
	RA2410	Female	
	RA1875	Male	90±2
	RA1853	Male	
	RA2360	Female	
	RA2467	Male	180±2
	RA2426	Male	
	RA2150	Female	
High dose (1.35 x 10^{13} GC)	RA2444	Male	14
	RA2468	Male	
	RA2363	Female	
	RA2457	Male	90±2
	RA2371	Female	
	RA2153	Female	
	RA2452	Male	180±2
	RA2400	Female	
	RA2375	Female	

Supplemental Table 1. Design of toxicology study for ICM administration of AAVhu68 expressing human SMN in adult NHPs.
Animals were randomized to treatment groups. All injections were performed in a total volume of 1 mL.

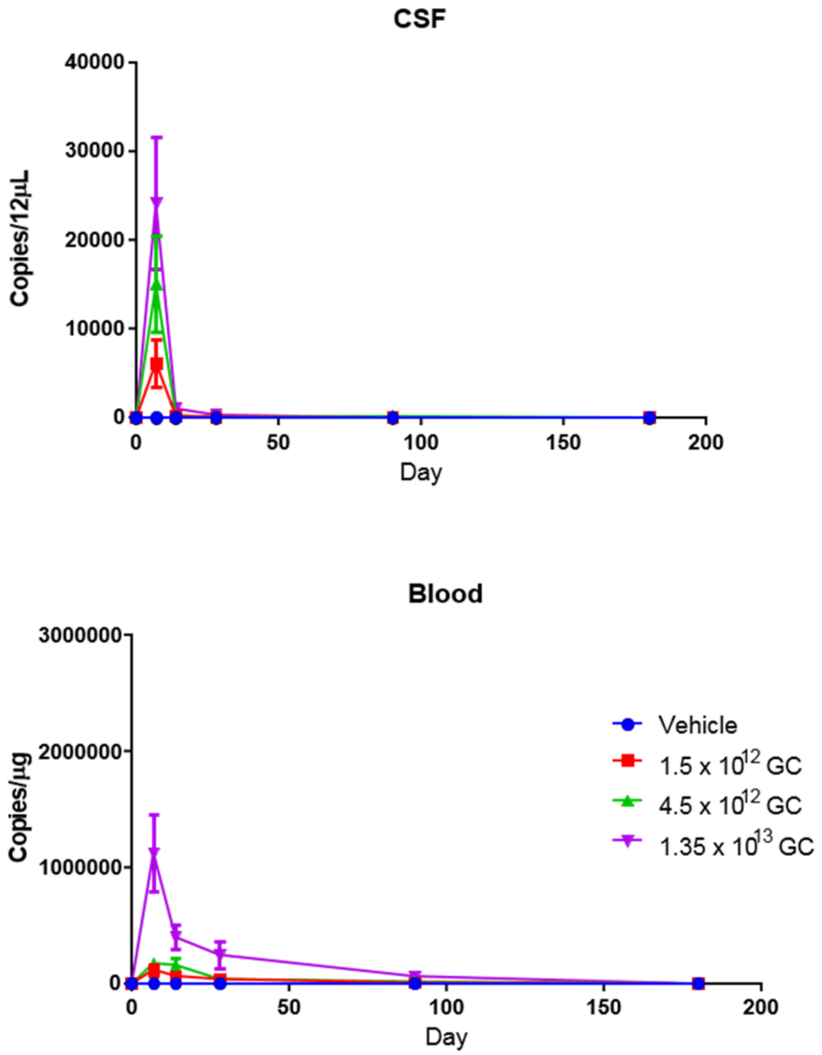

Supplemental Figure 1. Vector Genomes Measured in CSF and Blood Following ICM Administration to NHPs.

Each line represents mean vector genomes detected per microgram DNA (blood) or per 12 µL of CSF for each cohort. Error bars represent SEM.

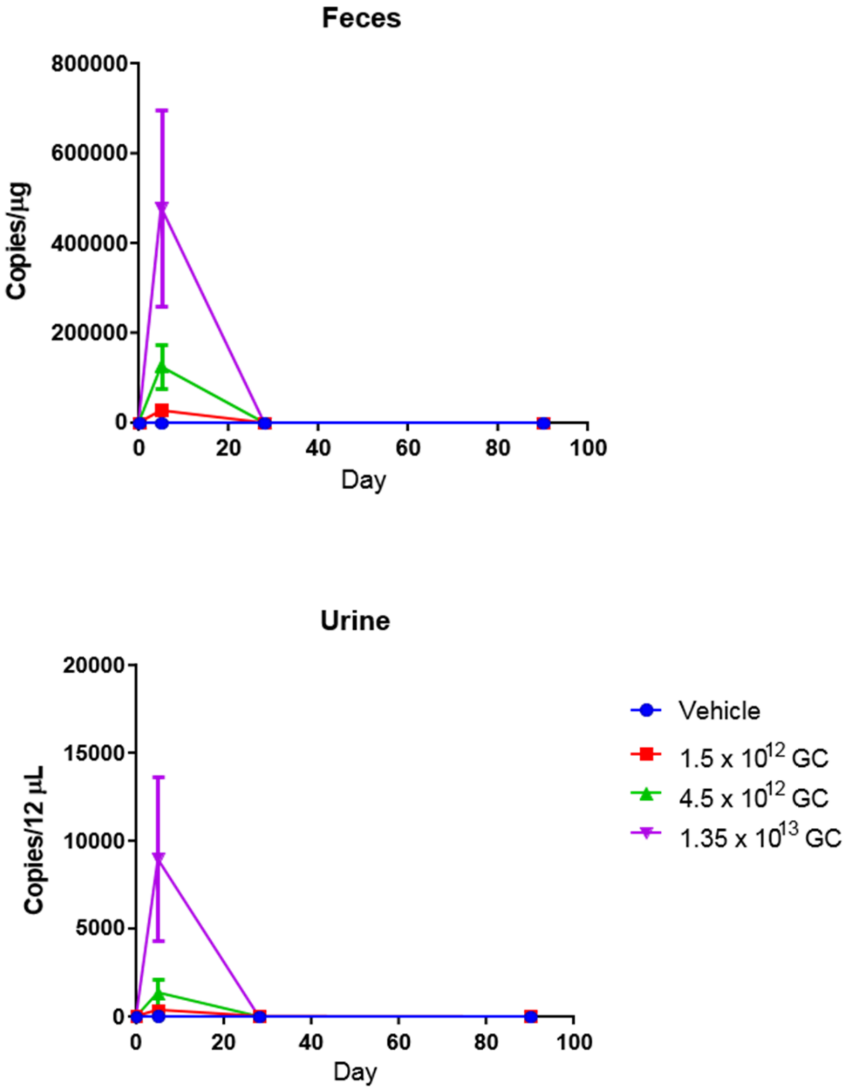

Supplemental Figure 2. Vector Excretion in Feces and Urine Following ICM Administration to NHPs

Each line represents mean vector genomes detected per µg DNA (feces) or per 12 µL of urine for each cohort. Error bars represent SEM.

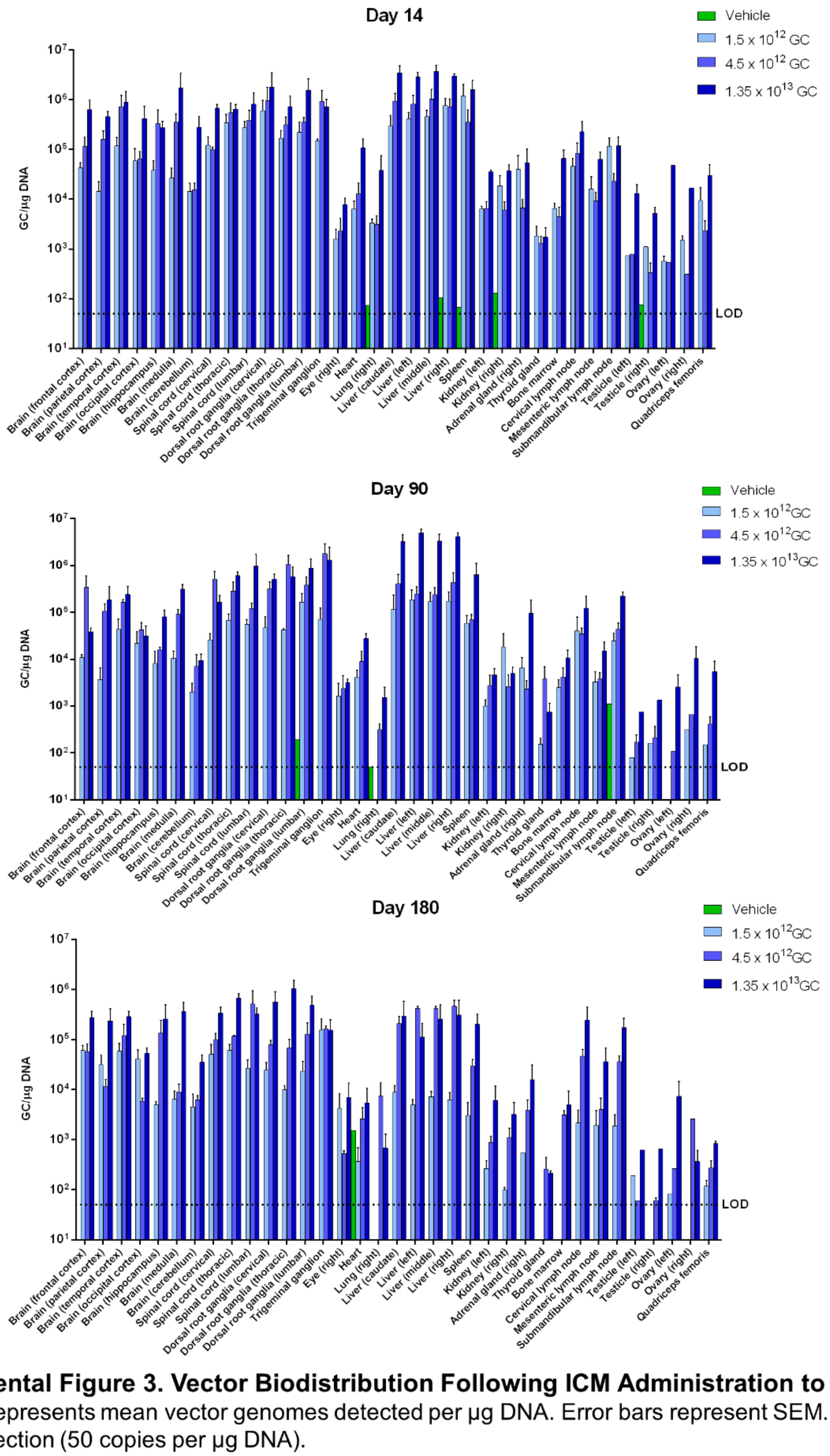

Supplemental Figure 3. Vector Biodistribution Following ICM Administration to NHPs. Each bar represents mean vector genomes detected per µg DNA. Error bars represent SEM. LOD = limit of detection (50 copies per µg DNA).

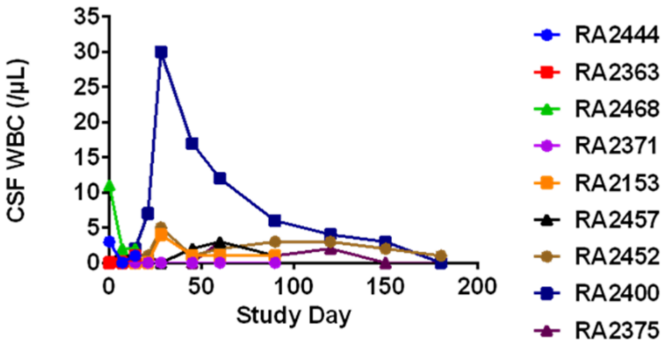

Supplemental Figure 4. CSF leukocyte counts in high dose cohort
AAVhu68 expressing human SMN was administered ICM to adult rhesus macaques at a dose of 1.35 x 10^{13} GC on Day 0. WBC = white blood cells.

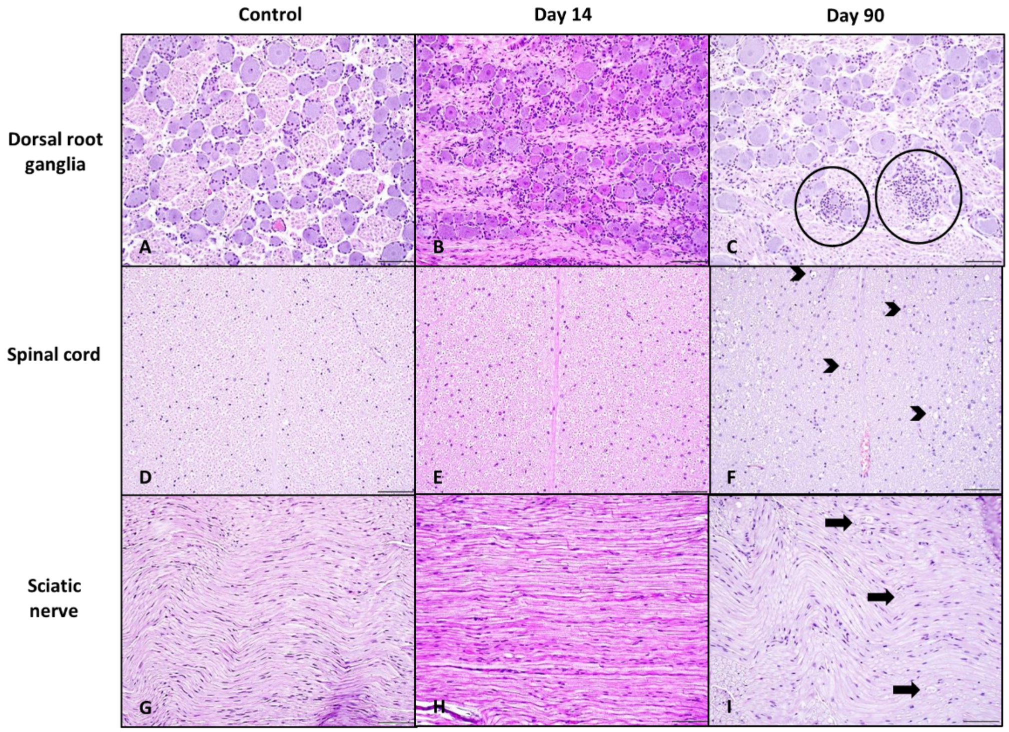

Supplemental Figure 5. Representative central and peripheral nervous system histopathologic findings in nonhuman primates administered AAVhu68 expressing human SMN via ICM injection. Pertinent vector-related findings were observed in the central and peripheral nervous system, primarily at the day 90 and day 180 time-point, with no dose effect. At the day 14 time-point, dorsal root ganglia (DRG) from a majority of vector-treated animals were histologically normal (A-B). Single neuronal degeneration was sporadically observed in few dorsal root ganglia segments and trigeminal ganglia of vector-treated animals as well as the trigeminal ganglia of the day 14 control animal; however, definitive interpretation of these findings were difficult due to limited number of control animals. No significant histologic findings were noted in the spinal cord (D-E) and peripheral nerves (shown: sciatic nerve; G-H) of vehicle control and vector-treated animals from the day 14 time-point. The dorsal root ganglia in few segments from treated groups at the day 90 time-point (C) had minimal neuronal cell body degeneration (circles) characterized by central chromatolysis, satellitosis and mononuclear cell infiltrates that surrounded and invaded neuronal cell bodies (neuronophagia). The majority of animals from all vector-treated groups had a minimal to moderate axonopathy of the dorsal white matter tracts of the spinal cord (F), which was bilateral and characterized by dilated myelin sheaths with and without myelomacrophages (arrowheads), consistent with axonal degeneration. The majority of these animals had a similar minimal to mild axonopathy (arrows) in peripheral nerves of both the fore- and hindlimbs (shown: sciatic nerves; I). A dose effect was not observed in any of the test article related findings. Findings at the day 180 time point were similar to those shown for Day 90, although severity was lower on Day 180. (Hematoxylin and eosin; Scale bar = 100 μm).

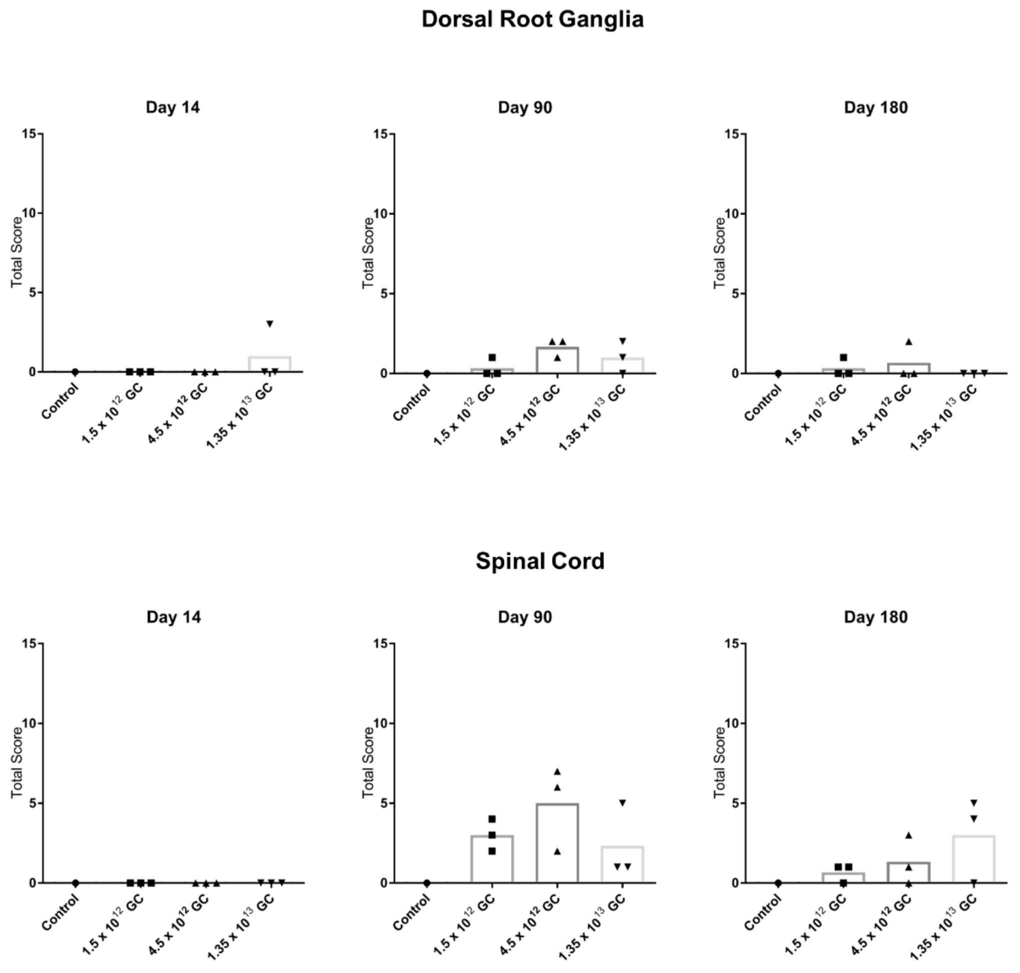

Supplemental Figure 6. Histological findings in primary sensory neurons of adult NHPs after ICM administration of AAVhu68 expressing human SMN.
A board-certified veterinary pathologist reviewed sections of dorsal root ganglia and spinal cord from the cervical, thoracic and lumbar levels. Neuronal cell body degeneration in dorsal root ganglia and axonal degeneration in the dorsal columns of the spinal cord were assigned a score of 0 (no findings present), or a severity score ranging from 1 (minimal) to 5 (severe). The total score was calculated for each animal by adding the score from the cervical, thoracic and lumbar levels. The maximum severity score possible is 15.

Dose (GC)	Animal ID	Necropsy Day	AAVhu68 NAb_{50}								
			Study Day								
			0	14	28	45	60	90	120	150	180
Vehicle	RA2424	14	5	< 5							
$1.5x10^{12}$	RA2031		< 5	160							
	RA1156		< 5	80							
	RA0704		< 5	160							
$4.5x10^{12}$	RA2463		5	20							
	RA2410		< 5	160							
	RA2433		< 5	80							
$1.35x10^{13}$	RA2444		< 5	160							
	RA2363		< 5	320							
	RA2468		< 5	160							
Vehicle	RA2456	90 ± 2	< 5	< 5	< 5	< 5	< 5	< 5			
$1.5x10^{12}$	RA2369		< 5	80	40	80	320	160			
	RA2412		5	160	160	320	640	80			
	RA0549		< 5	80	40	1280	640	320			
$4.5x10^{12}$	RA1875		< 5	80	80	2560	2560	2560			
	RA1853		< 5	160	320	1280	1280	2560			
	RA2360		< 5	80	160	2560	2560	320			
$1.35x10^{13}$	RA2371		5	40	40	1280	640	640			
	RA2153		< 5	160	320	2560	1280	1280			
	RA2457		< 5	40	160	2560	640	320			
Vehicle	RA2382	180 ± 2	< 5	< 5	< 5	< 5	< 5	< 5	< 5	< 5	< 5
$1.5x10^{12}$	RA2464		< 5	2560	320	1280	1280	1280	1280	1280	640
	RA2434		< 5	5	80	640	640	320	640	320	320
	RA2413		< 5	160	320	320	320	320	640	320	320
$4.5x10^{12}$	RA2467		< 5	160	160	640	640	320	320	320	160
	RA2426		< 5	80	640	640	640	640	1280	1280	320
	RA2150		< 5	160	80	640	1280	640	640	640	320
$1.35x10^{13}$	RA2452		< 5	80	40	160	640	160	320	160	320
	RA2400		20	160	1280	1280	2560	1280	1280	1280	640
	RA2375		5	160	160	640	1280	1280	1280	1280	640

Supplemental Figure 7. Neutralizing antibody responses to the vector capsid in serum of adult NHPs after ICM administration of AAVhu68 expressing human SMN.
The neutralizing antibody titer values reported are the reciprocal dilutions of serum at which in vitro cell transduction was reduced by at least 50% compared to control wells (without sample). The limit of detection was 1:5 dilution of sample. Day 0 samples were collected immediately prior to vector administration.

Dose (GC)	Necropsy Day	Animal ID	Antigen	PBMC Study Day								Liver	Spleen	Bone Marrow
				0	14	28	60	90	120	150	180			
Vehicle	14	RA2424	AAVhu68	-	-							-	-	-
			hSMN	-	-							-	-	-
1.5x10^{12}		RA2031	AAVhu68	-	-							+	-	-
			hSMN	-	-							+	-	-
		RA1156	AAVhu68	-	-							-	-	-
			hSMN	-	-							-	-	+
		RA0704	AAVhu68	-	-							-	-	-
			hSMN	-	-							-	-	+
4.5x10^{12}		RA2463	AAVhu68	-	-							-	-	-
			hSMN	-	-							-	-	-
		RA2410	AAVhu68	-	-							-	-	-
			hSMN	-	-							-	-	+
		RA2433	AAVhu68	-	-							-	-	-
			hSMN	-	-							-	-	-
1.35x10^{13}		RA2444	AAVhu68	-	-							-	-	-
			hSMN	-	-							-	-	-
		RA2363	AAVhu68	-	-							-	-	-
			hSMN	-	-							-	-	-
		RA2468	AAVhu68	+	+							-	-	-
			hSMN	-	-							-	-	-
Vehicle	90 ± 2	RA2456	AAVhu68	-	-	-	-	-				-	-	-
			hSMN	-	-	-	-	-				-	-	-
1.5x10^{12}		RA2369	AAVhu68	-	-	-	-	-				-	-	-
			hSMN	-	-	-	-	+				+	+	-
		RA2412	AAVhu68	-	-	-	-	-				+	-	-
			hSMN	-	-	-	-	-				-	-	-
		RA0549	AAVhu68	-	-	+	+	-				+	-	-
			hSMN	-	-	-	+	+				+	-	+
4.5x10^{12}		RA1875	AAVhu68	-	-	-	-	-				+	-	+
			hSMN	-	-	+	-	-				+	-	+
		RA1853	AAVhu68	-	-	-	-	-				-	-	-
			hSMN	-	-	-	-	-				-	-	-
		RA2360	AAVhu68	+	-	+	+	+				-	-	-
			hSMN	-	-	-	-	+				+	-	-
1.35x10^{13}		RA2371	AAVhu68	+	+	+	+	+				+	+	-
			hSMN	-	-	-	+	+				+	+	+
		RA2153	AAVhu68	-	-	-	-	-				+	-	-
			hSMN	-	-	-	-	-				-	-	-
		RA2457	AAVhu68	-	-	-	-	-				-	-	-
			hSMN	-	-	-	+	+				+	+	+
Vehicle	180 ± 2	RA2382	AAVhu68	-	-	-	-	-	-	-	-	-	-	-
			hSMN	-	-	-	-	-	+	+	-	-	-	-
1.5x10^{12}		RA2464	AAVhu68	-	+	+	+	-	+	-	-	-	-	-
			hSMN	-	-	-	+	-	-	-	-	-	-	-
		RA2434	AAVhu68	-	-	-	-	-	-	-	-	-	-	-
			hSMN	-	-	-	+	+	+	-	-	+	-	+
		RA2413	AAVhu68	-	-	-	-	-	-	-	+	+	-	-
			hSMN	-	-	-	-	-	-	-	-	-	-	-
4.5x10^{12}		RA2467	AAVhu68	-	-	-	-	-	-	-	-	-	-	-
			hSMN	-	-	-	-	-	-	-	-	-	-	-
		RA2426	AAVhu68	-	-	-	+	+	+	+	+	+	-	-
			hSMN	-	-	-	+	+	+	+	+	+	+	-
		RA2150	AAVhu68	-	-	-	-	+	-	-	-	-	-	-
			hSMN	-	-	-	-	+	+	+	-	-	-	-
1.35x10^{13}		RA2452	AAVhu68	-	-	-	-	-	-	-	-	-	-	-
			hSMN	-	-	-	-	-	-	-	-	-	-	-
		RA2400	AAVhu68	-	+	-	-	-	-	-	-	-	-	-
			hSMN	-	-	+	-	-	+	-	-	-	-	-
		RA2375	AAVhu68	-	-	-	-	-	+	+	-	-	-	-
			hSMN	-	-	-	-	-	-	-	-	-	-	-

Supplemental Figure 8. IFN gamma ELISPOT detection of T cell responses to AAVhu68 capsid and hSMN transgene. Lymphocytes were isolated from peripheral blood, bone marrow, liver and spleen. T cell responses were measured against pooled overlapping 15-mer peptides comprising the AAVhu68 VP1 sequence (3 peptide pools) or the human SMN sequence (2 peptide pools). + indicates a detectable T cell response (>55 spot forming units and 3-fold greater than unstimulated control) to the indicated antigen. PBMC, peripheral blood mononuclear cells.
